# Acid precipitation salting out as an efficient strategy for high purity potato trypsin inhibitor recovery

**DOI:** 10.1038/s41538-026-00912-6

**Published:** 2026-06-02

**Authors:** Lei Cai, Panchaonan Huang, Chun Cui

**Affiliations:** 1 School of Public Health and Health Management, Gannan Medical University, Ganzhou, Jiangxi China; 2 School of Food Science and Engineering, South China University of Technology, Guangzhou, Guangdong China

**Keywords:** Chemistry, Environmental sciences

## Abstract

Potato trypsin inhibitor (PTI) is a high-value bioactive protein from potato processing wastewater with potential applications in functional foods and nutraceuticals. This study systematically compared three extraction strategies—acid precipitation-salting out, direct resin adsorption, and zinc ion precipitation—for PTI recovery from simulated potato processing wastewater. Acid precipitation-salting out emerged as the most effective approach, yielding PTI with high purity (63.62% protein), superior specific activity (533.06 TIU/mg), and enhanced structural stability evidenced by high β-sheet content (48.09%), narrow particle size distribution, and elevated zeta potential. The extracted PTI exhibited favorable emulsifying capacity (66.68%), foam stability exceeding 80%, broad pH stability, and moderate resistance to simulated gastrointestinal digestion (73.98% activity retention). In contrast, resin adsorption achieved higher yield but lower purity and functionality, while zinc ion precipitation introduced significant heavy metal residue risks. This work establishes acid precipitation-salting out as a scalable strategy for high-quality PTI extraction and highlights its dual functionality as a bioactive and techno-functional ingredient, supporting sustainable valorization of potato processing waste.

**Figure:**
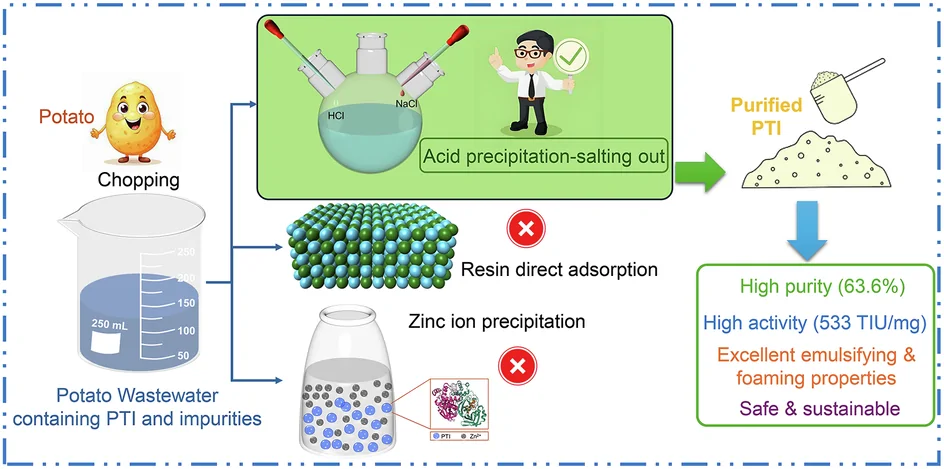

## Introduction

Potato trypsin inhibitor (PTI) is a well-studied protease inhibitor found in potato (*Solanum tuberosum* L.), renowned for its ability to inhibit trypsin, a key digestive enzyme in the human body^1^. The presence of PTI in potato-based foods has garnered significant attention due to its potential health implications, including antinutritional effects and potential therapeutic applications^2^. With the increasing demand for natural and functional food ingredients, the efficient extraction and purification of PTI from potato waste materials have become critical areas of research^2,3^. In recent years, with the advancement of scientific research, the physiological activities of trypsin inhibitors, including anticancer, antioxidant, antibacterial, and satiety-inducing effects, have garnered increasing attention^4–7^. These discoveries have revealed their broad application prospects in fields such as agricultural pest control and medical treatment. Therefore, developing novel extraction methods for trypsin inhibitors using potato starch processing wastewater as raw material holds significant economic value^8,9^.

Potato processing generates large amounts of wastewater containing a variety of valuable components, including proteins, sugars and other nutrients, with PTI being a potentially high-value by-product that can be recycled^3,10^. Efficient extraction and purification of PTI not only contributes to the valorization of potato waste but also provides a sustainable source of functional ingredients for the food and pharmaceutical industries^2,11^.

The extraction of PTIs from potato starch wastewater presents significant technical and economic challenges. Conventional methods, such as acid precipitation, often necessitate high equipment investment, yield low extraction efficiency, and can cause severe protein denaturation. This degradation results in the loss of functional and physiological properties, hindering scalable production and application in the food industry. In response, alternative methods have been explored, including salting out, adsorption using various materials, and metal ion precipitation^12^. Each technique offers a unique balance of extraction efficiency, product purity, and operational complexity. A critical barrier to the advancement and application of these methods is the lack of comprehensive, comparative studies. While existing research frequently focuses on optimizing a single extraction process - most commonly acid precipitation combined with salting out and ultrafiltration^11,13–16^ - the absence of parallel controls across different studies makes it difficult to objectively evaluate the overall efficacy of mainstream techniques such as acid precipitation, resin adsorption, and metal ion precipitation.

Furthermore, the characterization of extracted PTI in previous studies has been largely incomplete. Most existing work has focused on inhibitory activity retention under various conditions (pH, temperature, and simulated digestion), with limited attention to deeper structural properties such as secondary conformation, surface hydrophobicity, particle size distribution, and zeta potential - all of which are fundamental determinants of techno-functional performance (e.g., emulsification and foaming)^13^. Understanding these structure-function relationships is essential for predicting PTI’s behavior in complex food systems, yet such knowledge remains scarce.

In addition, a thorough assessment of PTI stability under previously unreported environmental conditions (e.g., various metal ions, light, oxygen) and a systematic evaluation of potential safety risks - particularly heavy metal residues from metal ion precipitation methods - are urgently needed but have been largely overlooked^1,12,17^. For instance, while Zn^2+^ precipitation has been explored for protein recovery in other contexts, no previous PTI study has quantitatively measured zinc residues in the final product or assessed its suitability for food-grade applications.

To address these research gaps, this study systematically compares and critically evaluates three primary extraction pathways-acid precipitation, salting out, direct resin adsorption, and zinc ion precipitation - for the first time under identical conditions using the same raw material and analytical platform. The innovation lies in an integrated research strategy combining method comparison, safety quantification, and structure-techno-function relationship analysis. Specifically, this research aims to: (1) clarify the comparative advantages of the three extraction pathways in terms of yield, purity, activity, and safety; (2) reveal how distinct extraction mechanisms influence PTI’s structural characteristics (secondary structure, particle size, zeta potential, surface hydrophobicity); (3) establish structure–activity relationships linking these structural features to techno-functional properties (emulsification, foaming, pH stability, metal ion stability, light/oxygen stability); and (4) provide a reliable and safe technical pathway for valorizing potato processing wastewater into a high-value dual-functional ingredient for the food industry.

## Results

### Protein and total sugar content of potato simulated processing wastewater

The simulated potato processing wastewater used in this study contained 0.53% protein and 1.30% total sugar (Table S1). These values correspond to protein and sugar accounting for 20.62% and 50.58% of the total solids content, respectively. These data were used for subsequent comparisons of changes in sugar content, protein content and calculation of purification multiplicity.

### Extraction of PTI by acid precipitation combined with salting-out

Potato wastewater is rich in a variety of protein fractions, including protease inhibitors, higher molecular weight glycoproteins and other macromolecular proteins^18^. The SDS-PAGE plots of the supernatant of potato simulated wastewater after different pH treatments are shown in Fig. S1A. As can be seen from the figure, the number of heteroprotein bands with higher molecular weights was significantly reduced after acid precipitation treatment, and the liquids extracted at different pH conditions contained trypsin inhibitors, which indicated that acid treatment had a significant effect on the removal of heteroproteins. It was reported that the retained bands under non-reducing electrophoresis conditions mainly corresponded to two trypsin inhibitors, PIII and KTI^15^. By comparing the bands of samples under different pH conditions (S2, S2.5, S3 with S3.5, S4, S4.5, and S5), it can be seen that the bands corresponding to glycoproteins as well as other macromolecules have disappeared and only the bands corresponding to trypsin inhibitors have been retained when the pH of the wastewater was 2, 2.5 or 3, which suggests that heteroproteins can be effectively removed when the pH of the acid precipitation was set to 2, 2.5 or 3.

The effect of different acid precipitation conditions on protein recovery and inhibitor activity recovery can be seen in Fig. S1C. The results showed that the protein recovery of the sample solution pH 2.0 to pH 3.0 was significantly low, and there was a significant difference compared with the pH 3.5 group (*p* < 0.05). However, there was no significant change in the activity recovery (*p* > 0.05), suggesting that when the pH of acid precipitation was adjusted to 2–3, more heteroproteins that did not have trypsin inhibitor activity were removed, resulting in lower protein recoveries, while the trypsin inhibitor was retained. This may be due to the significant difference in the isoelectric point distributions of potato glycoproteins and trypsin inhibitors, with the former ranging from 4.5 to 5.2 and the latter from 5.1 to 9.0.

Based on this property, when the pH of the solution is adjusted to a condition close to the isoelectric point of glycoprotein but far from the isoelectric point of trypsin inhibitor, the glycoprotein will be more prone to sedimentation, while the trypsin inhibitor will remain in a relatively stable solubility state due to its higher isoelectric point. Secondly, given that PTIs are known to usually possess strong acid resistance, when the experimental conditions are set at a lower pH, proteins other than trypsin inhibitors may lose activity due to the increase in ambient acidity, which in turn results in the formation of precipitates^11^. Further comparisons of the results revealed that S3 had the highest activity recovery. Therefore, pH 3 was used as the optimal acid precipitation condition for subsequent experiments. Similar results were obtained in the report of Li et al.^13^.

In the study of protein separation, the salt precipitation method is popular because of its mild conditions, easy operation and low cost. The precipitation of proteins is achieved by a dual mechanism: on the one hand, the water film formed on the surface of protein colloidal particles is effectively disrupted by reducing the hydrogen bonding between the protein and water molecules, which significantly weakens the stability of proteins in water. On the other hand, the solubility of proteins is further reduced by the introduction of neutral salts that substantially neutralize the charge on protein molecules^11^. Together, these two effects contribute to the precipitation of proteins from solution, which leads to the separation of proteins.

According to Fig. S1B, Y1 to Y5 had bands between 15 and 25 kDa, while no significant bands were seen in the supernatants of Y6 to Y9. This phenomenon indicates that the addition of (NH_4_)_2_SO_4_ was not effective in promoting the precipitation of PTI. It is worth noting that with the gradual increase of NaCl concentration, the color of protein bands in the supernatants of Y1 to Y5 showed a tendency of fading and then deepening, which further corresponded to the phenomenon of increasing and then decreasing the amount of PTI precipitation. In contrast, (NH_4_)_2_SO_4_ did not significantly affect the electrophoretic bands of PTI at four different concentrations. As can be seen from Fig. S1D, although (NH_4_)_2_SO_4_ effectively promoted protein precipitation during salting-out, NaCl was much more effective in recovering the inhibitory activity of PTI, which was up to 82.5%. This phenomenon indicated the lower selectivity of (NH_4_)_2_SO_4_ for the salting-out of PTI, which led to the recovery of other heterogeneous proteins, resulting in a higher protein recovery, compared with the higher specificity of NaCl for PTI precipitation^13^. In summary, 35% NaCl was used as the preferred salting-out condition for recovering PTI from wastewater. Figure S1E shows the SDS-PAGE graph of PTI prepared under these conditions, from which there was only one protein band between 15 and 25 kDa and no other heterogeneous proteins, indicating a better separation.

### PTI extraction by direct adsorption method

The protein adsorption rate of potato simulated wastewater after adsorption by different adsorption materials is shown in Fig. S2A. As can be seen from the figure, the protein adsorption rate of the 16 materials increased with the increase of the material-liquid ratio, and the resin with the highest protein adsorption rate was the XDA-1 resin at the material-liquid ratio of 2:5, which was 71.02%. The protein adsorption effect of D-101 resin~732 anionic resin (Na) on protein adsorption of potato wastewater was poorer, especially the protein adsorption rate of the 732 anionic resin (Na) was lower than 5%.

The inhibitory activity adsorption rate of potato simulated wastewater after adsorption by different materials is presented in Fig. S2B. As can be seen from the figure, the adsorption rate of inhibitory activity of the materials was low when the material-liquid ratio was 1:10, and when the material-liquid ratios were 1:5 and 2:5, the adsorption rate of inhibitory activity was as high as 85% to 90%, among which the highest adsorption rate was 92.47% for the XAD-16 resin at the material-liquid ratio of 2:5. The adsorption rate of hydrated silica on inhibitory activity was lower compared with other resins, 39.45% at the material-liquid ratio of 1:10.

Figure S2C illustrates the SDS-PAGE of the remaining proteins of potato wastewater after adsorption by different materials. The lighter color of the bands represents the stronger adsorption capacity of the material for the protein in potato wastewater, and the color of bands 4, 5, 6, 10 and 14 is very light, which indicates the strong adsorption capacity of its resins AB-8, LX-8, HPD-400, HPD-600, and hydrated silica for potato protein. Bands 3, 12 and 13 are very dark and not much different from the representative potato wastewater bands 1 and 9, which indicates that after adsorption of potato wastewater by the resin, there is not much difference between the residual protein content and the protein content in the potato wastewater, i.e., the adsorption capacity of the resins ADS-600, LXTE-600, and LXTE-603 for potato proteins is weak. Bands 2, 7 and 11 were only light in the protease inhibitor part, and the other protein molecular weight parts still had bands, which indicated that their resins HPD82, XDA-1, and XAD-16 specifically adsorbed PTI, especially the resin XAD-16. Therefore, in combination with the results of the specific adsorption of potato protein by the resins and the adsorption capacity of the protein and inhibitory activity, the resins LX-8, XAD-16 and XAD7HP were used for subsequent desorption experiments.

The best-performing resins (LX-8, XAD-16, and XAD7HP) share favorable properties for PTI adsorption: high surface area, appropriate pore size (compatible with ~20 kDa proteins), and suitable polarity (non-polar to weakly polar) that promotes hydrophobic and hydrogen-bonding interactions with PTI. In contrast, resins with mismatched polarity or pore size (e.g., ADS-600, LXTE-600) showed poor adsorption.

The plots of protein desorption rate and activity recovery for different types and concentrations of desorption solutions are shown in Fig. S2D, E. The maximum desorption rate was 26% for resin XAD-16 after desorption by 30 mM NaOH solution. As can be seen from the graphs, NaOH solution had the lowest protein desorption rate for the three adsorption resins, and the largest desorption rate was 26.8% for resin XAD-16 after desorption by a concentration of 30 mM NaOH solution, and especially its inhibitory activity recovery was extremely low, with a maximum value of 0.67%. However, 1% to 5% NaCl solution, methanol solution, and ethanol solution showed higher protein desorption rates for the three adsorption resins. Specifically, for resin LX-8, the highest protein desorption rate condition was 3% NaCl, with a desorption rate of 61.0%; whereas for resin XAD-16, the highest protein desorption rate condition was 80% methanol, with a desorption rate of 70.4%, which was the highest among all the sample groups; and for resin XAD7HP, the highest protein desorption rate condition was 5% NaCl, with a desorption rate of 54.6%. Additionally, similar to the NaOH solution group, the recovery of inhibitory activity for the NaCl solution was extremely low, with a maximum value of only 1.64%. The recoveries of inhibitory activity for both methanol and ethanol solutions were not high, and the maximum recovery for the methanol group was 8.19% after desorption of resin XAD-16 by a methanol solution of 40% concentration. The recovery of inhibitory activity of ethanol solution for the three adsorbent resins was the highest among all desorption solutions, and the maximum recovery was 15.7% for resin XAD-16 after desorption by 60% ethanol solution. Therefore, combining the results of protein desorption rate as well as activity recovery, the resin XAD-16 was finally determined as the adsorbent resin, and the desorption solution was 60% ethanol.

Resin XAD-16 was able to specifically adsorb PTI, and its inhibitory activity recovery was higher relative to other resins, and the ratio of inhibitory activity recovery to protein recovery was the largest, which indicated that its purification effect was the best. Figure S2F is the SDS-PAGE graph of the resin XAD-16 after the adsorption was completed and desorbed by different concentrations of ethanol. The protein molecular weight of the desorbed product is concentrated at 15–20 kDa, indicating that it is PTI, and there are no other heterogeneous proteins in the bands, which proves that the separation effect of this method is better. The greatest purification effect of XAD-16 can be justified by its material properties. XAD-16 showed the greatest purification effect due to its non-polar, macroporous structure and high surface area (~800 m^2^/g). These properties promote strong hydrophobic interactions with PTI (~20 kDa) while excluding larger glycoproteins and smaller impurities. In contrast, resins with mismatched polarity or pore size (e.g., LXTE-600, ADS-600) showed poorer selectivity.

### Extraction of PTI by metal ion precipitation method

**Fig. 1:**
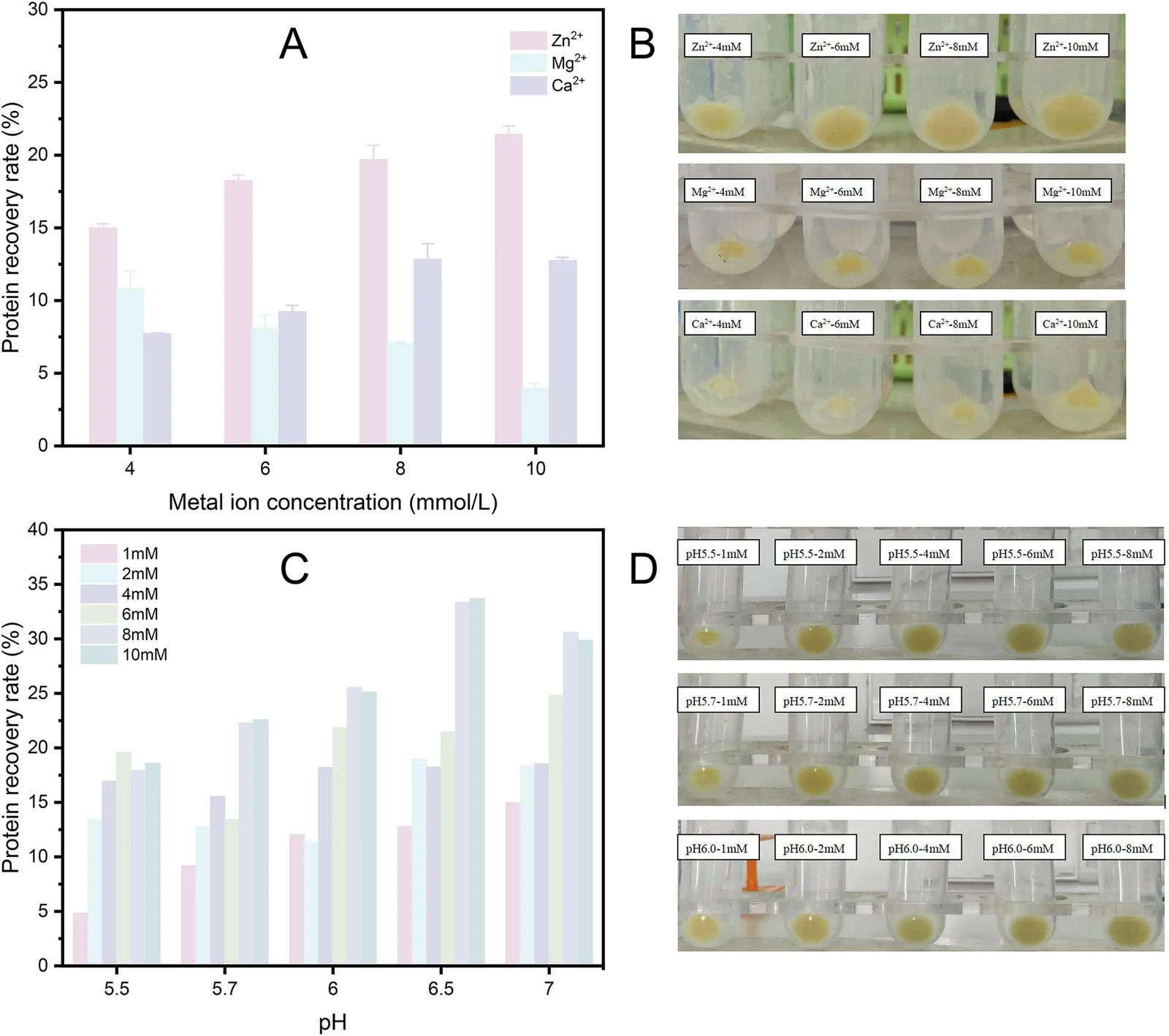
Effect of metal ions and pH on protein recovery and precipitation from potato wastewater. **A** Effect of different metal ions (Ca^2+^, Mg^2+^, Zn^2+^) on protein recovery. **B** Effect of different metal ions (Ca^2+^, Mg^2+^, Zn^2+^) on protein precipitation. **C** Effect of pH on protein recovery using different concentrations of Zn^2+^. **D** Effect of pH on protein precipitation using different concentrations of Zn^2+^.

Figure 1 demonstrates the effect of three metal ions on protein precipitation of potato wastewater. From the figure, Zn^2+^ has the best effect on protein precipitation and higher protein recovery. From Fig. 1A, the protein recovery increased with the increase of Zn^2+^ concentration, which was 22.44% when the Zn^2+^ concentration was 10 mM. The trend of Ca^2+^ group was similar to that of Zn^2+^ group, but the protein recovery was lower. In contrast, the protein recovery decreased with increasing Mg^2+^ concentration, which may be because the protein recovery of the Mg^2+^ group reached its maximum value before 4 mM concentration, probably because Mg^2+^ is more sensitive to potato waste liquid protein. Further combined with Fig. 1B, under the same conditions, Zn^2+^ was the most effective for protein precipitation, which produced the most precipitation and higher protein recovery, so zinc ions were subsequently selected for the next experiment. Zhang et al.^19^ revealed that metal ions (Al^3+^, Fe^2+^, Cu^2+^, and Zn^2+^) could bind to Penta-O-galloyl-β-d-glucopyranose (PGG) to form a PGG-metal complex and interact with proteins to form a PGG-metal-protein ternary complex, which resulted in the precipitation of PGG-protein increase.

As can be seen from Fig. 1C, in the range of pH 5.5–7, the protein recovery of different concentration groups had an increasing tendency with the increase of pH. Moreover, under the same pH condition, the protein recovery displayed a tendency to increase and then remain unchanged with the increase of Zn^2+^ concentration. The protein recovery reached the maximum value of 33.69% at pH 6.5 and Zn^2+^ concentration of 10 mM. Further analysis reveals that the results of Fig. 1C coincide with those of Fig. 1D in that the amount of precipitation becomes more as the Zn^2+^ concentration increases. Deng et al.^20^ found that low concentrations of K^+^ significantly improved gel properties, while Ca^2+^, Zn^2+^ and Fe^3+^ were more favorable to the formation of protein polymers, thus affecting the properties of alkali-induced egg white gels through the formation or disruption of intermolecular forces.

In Fig. S3A, the inhibitory activity recovery of potato wastewater increased with an increase of Zn^2+^ concentration and reached a maximum value of 40.16% at a Zn^2+^ concentration of 6 mM. With further increase in concentration, the inhibitory activity recovery showed a decreasing trend, which may be due to the inhibitory effect of excess metal ions on the inhibitory activity of trypsin inhibitor. There was no significant difference in the recovery of inhibitory activity at Zn^2+^ concentrations of 6 mM and 8 mM. Further analysis of the results in combination with Fig. 1C, D revealed that the protein recovery at 6 mM was lower than that at 8 mM, which suggests that Zn^2+^ at 8 mM may precipitate more heterogeneous proteins from potato wastewater, which is not conducive to the purification of PTI, and therefore 6 mM of Zn^2+^ was chosen as the final condition. Zn^2+^ showed the best protein precipitation performance, with maximum recovery at pH 6.5 and 6 mM Zn^2+^. The pH dependency reflects enhanced Zn^2+^-protein coordination at higher pH due to deprotonation of binding groups. However, Zn^2+^ concentrations above 6 mM reduced inhibitory activity recovery, suggesting excess metal ions may induce partial PTI denaturation. There was no significant difference in the color of the protein bands of potato wastewater after precipitation with different concentrations of Zn^2+^ in Fig. S3B, which may be due to the fact that the amount of protein precipitation produced during precipitation was small, causing the SDS-PAGE plots to show no difference^13^.

Furthermore, potato wastewater, potato wastewater supernatant after Zn^2+^ precipitation and precipitated freeze-dried powder were subjected to zinc ion content determination. Due to the excessively high Zn^2+^ residues in the final product (Table S2), the Zn^2+^ precipitation method was excluded from subsequent structural and functional characterizations.

### Characterization analysis of PTI

**Fig. 2:**
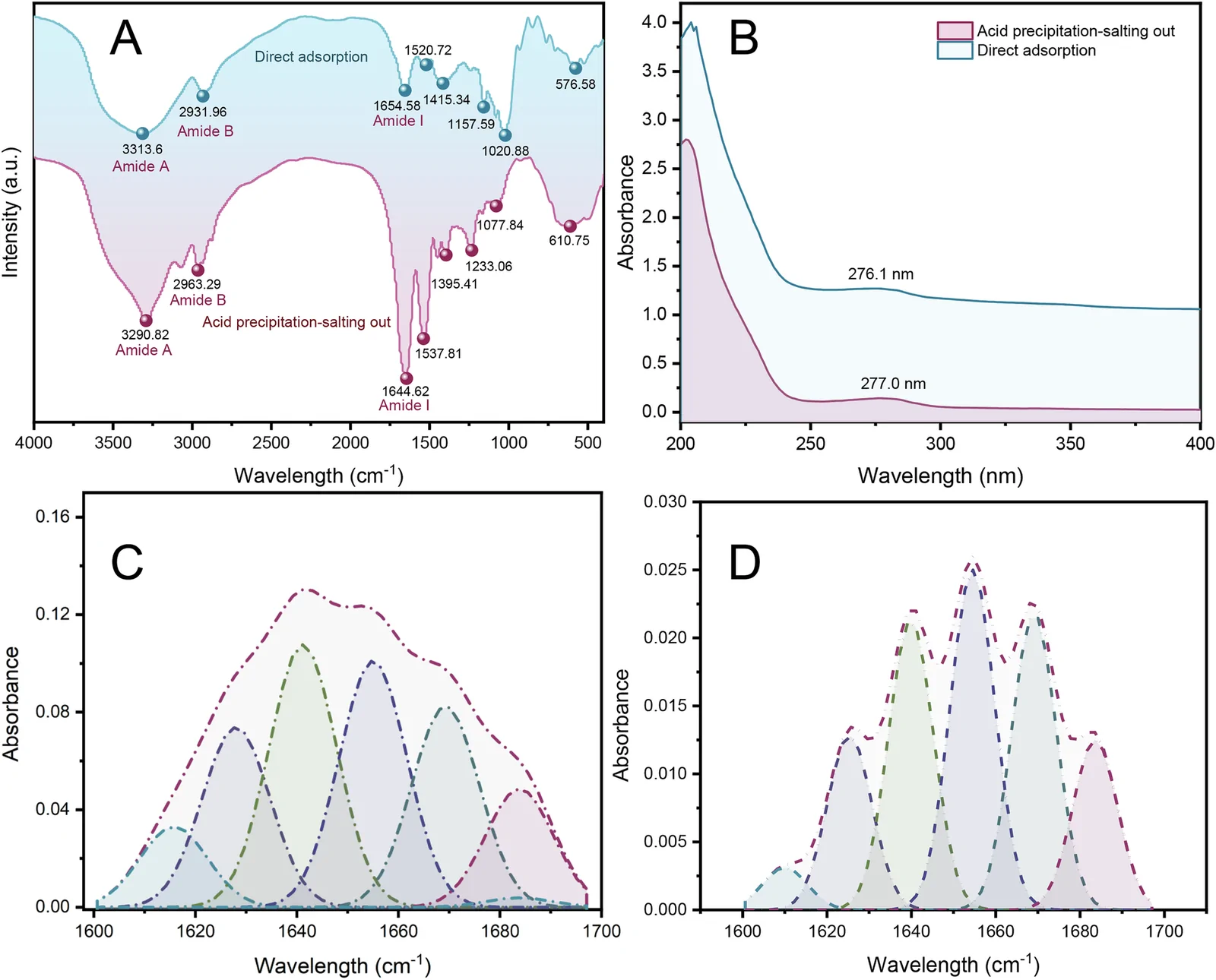
Spectroscopic characterization of potato trypsin inhibitor (PTI) prepared by two methods: acid precipitation combined with salting out (AP+SO) and direct adsorption (DA). **A** FTIR spectra of products from AP+SO and DA. **B** UV absorption spectra of products from AP+SO and DA. **C** Amide I region of FTIR spectra fitted for the AP+SO product. **D** Amide I region of FTIR spectra fitted for the DA product.

The following characterization applies only to PTI prepared using the two methods deemed safe for food applications (acid precipitation-salting out and direct resin adsorption). Fourier Transform Infrared Spectrometer (FTIR) spectroscopy has become a commonly used protein structure analysis tool to characterize the secondary structure of proteins. Variations in the amide I and amide III bands are closely related to the secondary structure of proteins, with the amide I band (1600–1700 cm^−1^) pair being widely used to study the secondary structure of proteins^21^. Therefore, the corresponding characteristic absorption bands can be processed by Gaussian fitting, deconvolution, and second-order derivatives to infer the secondary structure of the prepared PTI. The characteristic absorption bands 1537 cm^−1^ and 1395 cm^−1^ for the peptide chain functional group -H-N-C=O correspond to the absorption peaks of the trans and cis configurations of -H-N-C=O, respectively^22–26^. As shown in Fig. 2A, the intensity of the absorption peak at 1537 cm^−1^ was higher than that at 1395 cm^−1^ for the product prepared by acid precipitation combined with salting out, while the opposite was observed for the direct adsorption method, indicating that the product prepared by acid precipitation combined with salting out was more stable than that by direct adsorption, which might be due to the fact that it had not been damaged during the extraction to maintain its natural conformation.

The signals at the amide A band (3700~3200 cm^−1^) were mainly caused by the N–H and O–H stretching vibration, and both products showed strong absorption peaks, but the peak widths and peaks were quite different, suggesting that the different preparation methods may cause the breaking or increasing of the hydrogen bonds in the proteins, which is significant for the maintenance of the secondary structure of the proteins^24,27^. The signal at the amide B band (3000~2800 cm^−1^) was mainly caused by C–H stretching vibration^24^. Absorption peaks of low intensity appeared in both products, in which the peak value of the product prepared by the direct adsorption method was 2931.96 cm^−1^, differing greatly from that of the product prepared by acid precipitation combined with the salting out method at 2963.29 cm^−1^, which suggests that the different preparation methods have an effect on the C-H vibration in the product proteins, which may affect their hydrophobicity^28^.

The amide I band (1600~1700 cm^−1^) is generally considered in the infrared spectral analysis of proteins to be associated with the characteristic C–O stretching vibration, which is closely related to the secondary structure of proteins^29^. The different wavelength ranges (1600~1640 cm^−1^), (1640~1650 cm^−1^), (1650~1660 cm^−1^), and (1660~1700 cm^−1^) within the amide I band correspond to the different types of secondary structure of the protein, such as β-folding, random coiling, α-helix, and β-turns, respectively^24^. The amide I bands were analyzed by Gaussian curve fitting using Peakfit software (PeakFit v4.12, SeaSolve Software Inc., USA) to accurately quantify the relative amount of secondary structure in the products. The results are summarized in Table 1 and in Fig. 2C, D. The secondary structures of both products were dominated by β-structures, with β-folding and β-turning accounting for 48.09% and 28.95%, respectively, in the product of the acid precipitation combined with the salting-out method, whereas the product of the direct adsorption method had a higher proportion of β-turning (40.38%) and β-folding accounted for 22.00%. β-folding includes both parallel and antiparallel structures, which are more important in maintaining protein stability than β-turns^13,30^. Therefore, the products prepared by acid precipitation combined with the salting-out method showed better protein stability.

**Table 1:** Secondary structure composition of samples obtained with different methods

Sample	β-folding (%)	irregular curling (%)	α-helix (%)	β-turns (%)
Acid precipitation-salting out	48.09	15.34	7.62	28.95
Direct adsorption	22.00	19.54	18.08	40.38

Figure 2B lists the UV absorption spectra of the two PTI products scanned in the range of 210~400 nm. The product prepared by acid precipitation combined with salting out had an absorption peak at 277.0 nm, and the one by direct adsorption had an absorption peak at 276.1 nm, indicating that the purified protein had the characteristic peak of aromatic amino acid residues (280 nm) that are generally found in proteins^31^.

**Fig. 3:**
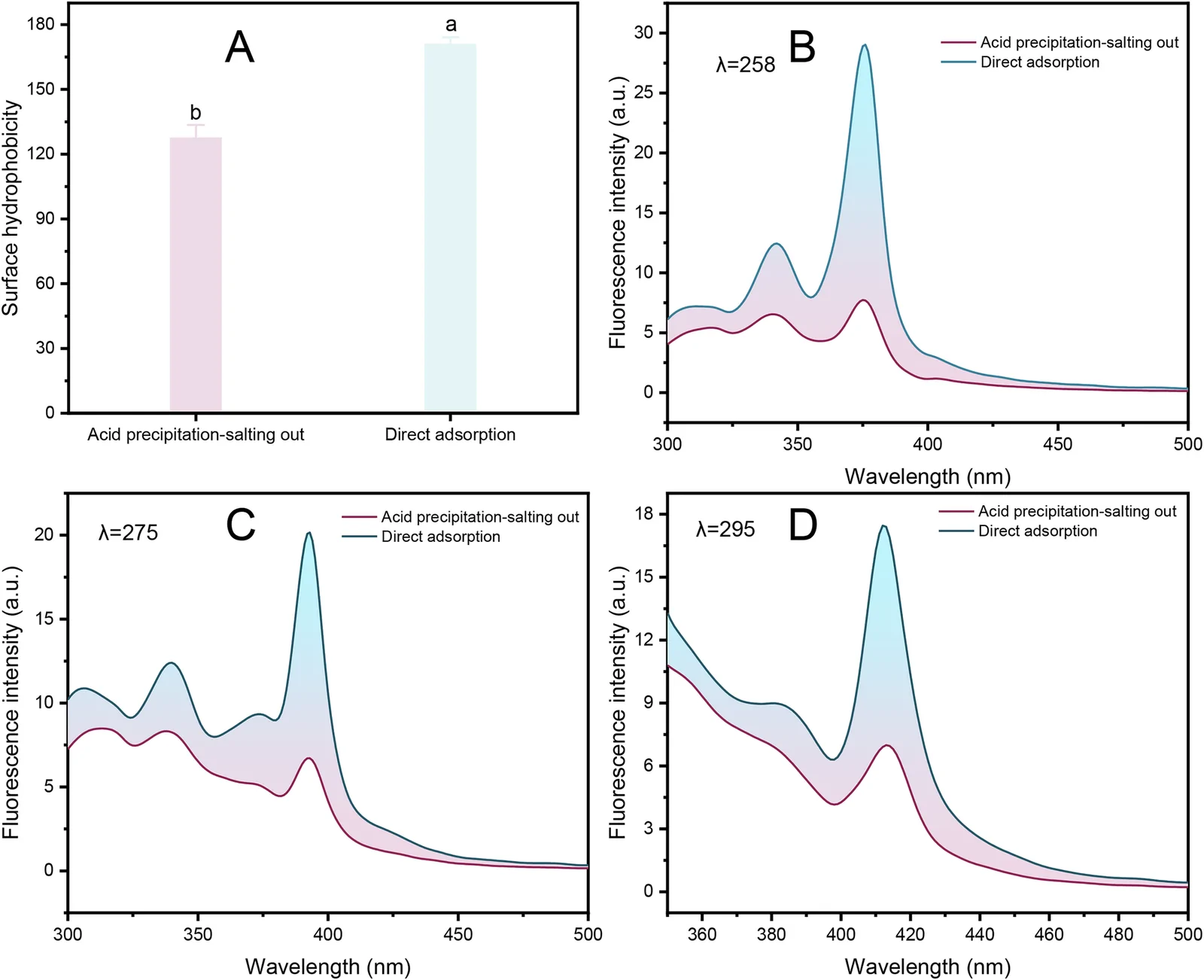
Surface hydrophobicity and endogenous fluorescence of PTI products from the two preparation methods. **A** Surface hydrophobicity. **B** Endogenous fluorescence at excitation λ = 258 nm. **C** Endogenous fluorescence at excitation λ = 275 nm. **D** Endogenous fluorescence at excitation λ = 295 nm. Different letters indicate significant differences (*p* < 0.05).

The surface hydrophobicity of the two product proteins was plotted in Fig. 3A. The surface hydrophobicity properties of the proteins are considered to be closely related to the distribution of hydrophobic amino acid residues on their surfaces^32^. The surface hydrophobicity of the product of the direct adsorption method was significantly higher than that of the product of acid precipitation combined with the salting-out method, which may be because the adsorption of the resin led to the exposure of some hydrophobic structural domains (such as hydrophobic amino acid residues, etc.) of the inhibitor protein. The FTIR results (Table 1) correlate well with surface hydrophobicity (Fig. 3A). The acid precipitation-salting out product, with higher β-sheet content (48.09%), showed lower surface hydrophobicity because β-sheets bury hydrophobic residues inside the protein core. In contrast, the direct adsorption product, with lower β-sheet and higher β-turn content (40.38%), exhibited higher surface hydrophobicity, suggesting that resin treatment induced partial unfolding and exposure of hydrophobic domains.

There exists an intrinsic fluorescence property in the protein structure that mainly originates from specific amino acid residues within their molecules, including the aromatic structures of tryptophan (Trp), tyrosine (Tyr) and phenylalanine (Phe), which have specific fluorescence excitation wavelengths of 295, 275 and 258 nm, respectively^33^. These amino acid residues can emit fluorescence at specific wavelengths, and their fluorescence properties (including the maximum fluorescence wavelength *λ*_max_ and fluorescence intensity) are extremely sensitive to changes in the polarity of the microenvironment in which they are found. Therefore, they are widely applied in characterizing the dynamic changes of protein tertiary structure^34^. As shown in Fig. 3B–D, like the surface hydrophobicity results, the fluorescence intensity of the samples from the direct adsorption method was higher than that of the acid precipitation combined with the salting out method, which may be attributed to the adsorption of the resin, resulting in the exposure of the aromatic amino residues of the protein.

**Fig. 4:**
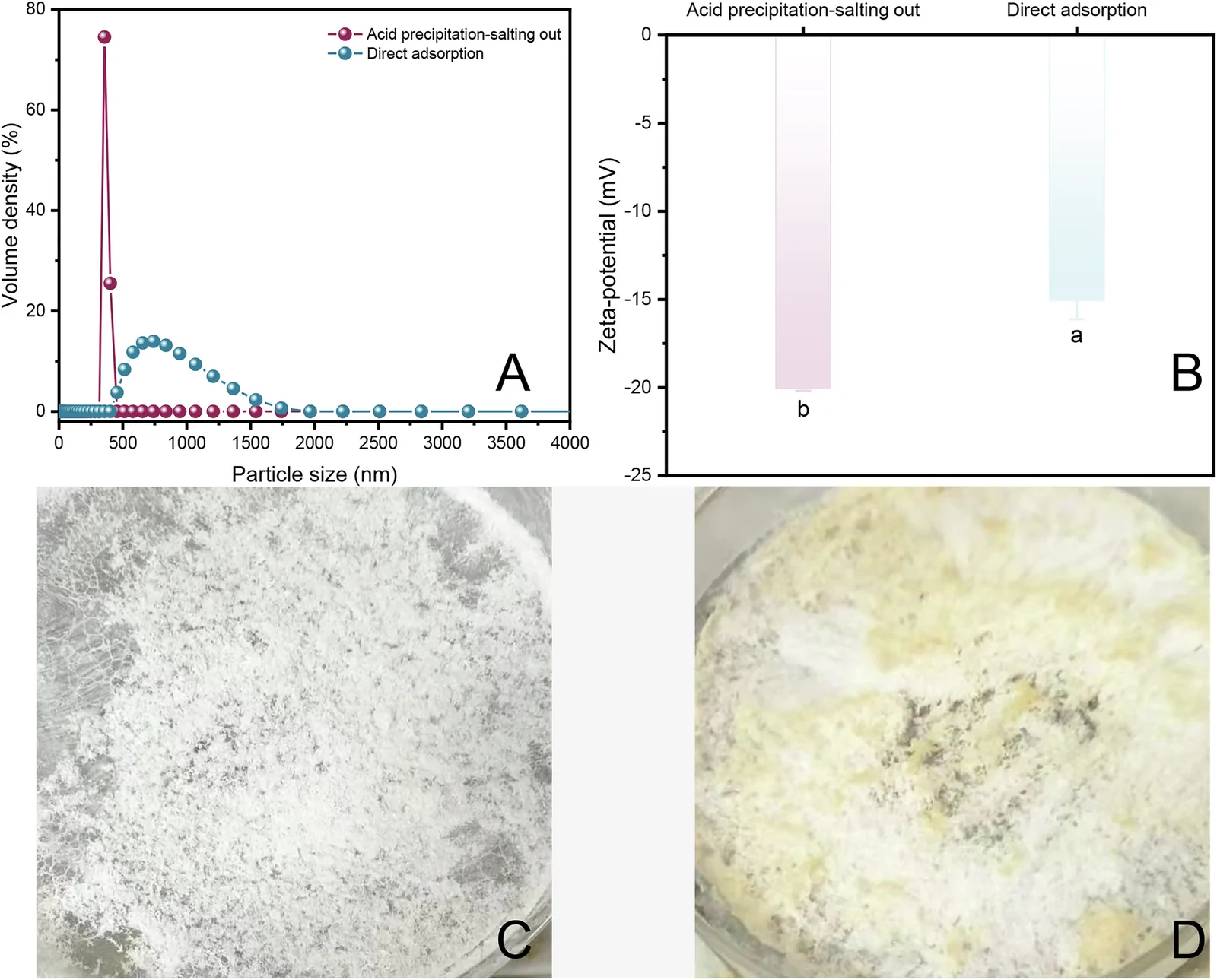
Particle size, zeta potential, and appearance of PTI products from the two preparation methods. **A** Particle size distribution. **B** Zeta potential. **C** Appearance of the product from acid precipitation combined with salting out. **D** Appearance of the product from direct adsorption. Different letters indicate significant differences (*p* < 0.05).

Particle size is an important characterization index of proteins, indicating the size of protein particle diameter. The particle sizes of the sample proteins prepared by different methods are shown in Table S3 (Supplementary Material) and Fig. 4A. The particle size of PTI obtained by acid precipitation combined with salting out was smaller and more concentrated in distribution than that obtained by direct adsorption. In general, a small particle size results in a large specific surface area, which enhances the interaction between the protein and water molecules and has a greater influence on the properties of the protein. d (0.05), d (0.1), d (0.5) and d (0.9) are the four parameters corresponding to the size values measured for 5%, 10%, 50% and 90% of the particle sizes in the particle-size distribution diagram, respectively.

The electrostatic repulsion between molecules when the protein is dispersed in the solvent plays an important role in maintaining the dispersion stability of the solution^35^. Zeta potential represents the potential difference between the surface of the solid particles and the solvent, which reflects the surface electrical properties of the particles in the solution. The higher absolute value of the zeta potential indicates the stronger intermolecular repulsion, less aggregation and better stability of the system^36^. Figure 4B reveals that the absolute value of zeta potential of the sample prepared by acid precipitation combined with the salting out method is significantly higher than that of the direct adsorption method, which indicates that the PTI prepared by acid precipitation combined with the salting out method has higher stability.

Table 2 provides a comparison of the optimization results of the three preparation methods: adsorption on macroporous resin, acid precipitation combined with salting out and metal ion precipitation. From the results, the PTI obtained by acid precipitation combined with the salting out method has high protein content, low sugar content, high inhibitory activity and greater purification times. Although the PTI obtained by direct adsorption method had a high yield, the sugar content was high and the purification factor was too low, which was due to the fact that the resin would not only adsorb the target proteins, but also adsorb the sugar-containing substances such as glycoproteins in the potato wastewater and these sugar substances would be desorbed out during the desorption process resulting in a high sugar content in the samples.

**Table 2:** Comparison of samples obtained by different methods

Samples	Protein content (%)	Sugar content (%)	Yield (mg/kg)	Inhibitory activity (TIU/mg)	Purification factor
Simulated wastewater	20.62 ± 0.65	50.74 ± 1.68	-	47.17 ± 0.12	1
Acid precipitation-salting out	63.62 ± 2.13	8.90 ± 0.29	933 ± 39	533.06 ± 6.48	11.30
Direct adsorption	40.69 ± 1.66	20.69 ± 0.81	1287 ± 54	72.98 ± 0.27	1.55
Metal ions precipitation	57.36 ± 1.89	17.52 ± 0.63	1250 ± 58	175.52 ± 12.57	3.72

Besides, the resin also adsorbs pigments, and the final PTI obtained was not only yellowish in color but also sticky in texture (Fig. 4D), whereas the sample powder obtained by acid precipitation combined with the salting out method was pure white (Fig. 4C) and dry in texture. Therefore, the PTI prepared by acid precipitation combined with the salting out method was chosen for the subsequent experiments. The above positive changes indicated that all the adopted PTI preparation methods effectively extracted the target proteins and significantly improved the trypsin inhibitory activity. Sugars usually bind to proteins with non-covalent bonds to form glycoprotein complexes, and the significant reduction in total sugar content could be attributed to the removal of glycoproteins during the PTI extraction and purification process^37–39^. Ji et al.^40^ combined resin adsorption, pH adjustment, salt/ethanol precipitation, and ultrafiltration to isolate lysozyme, ovalbumin, ovalbumin, and ovalbumin sequentially from egg white by FPC 3500 resin. The purification process resulted in a purity of more than 90% (about 72% for ovalbumin) and a yield of more than 77% (about 75% for ovalbumin) for each protein sample.

### Solubility of PTI

**Fig. 5:**
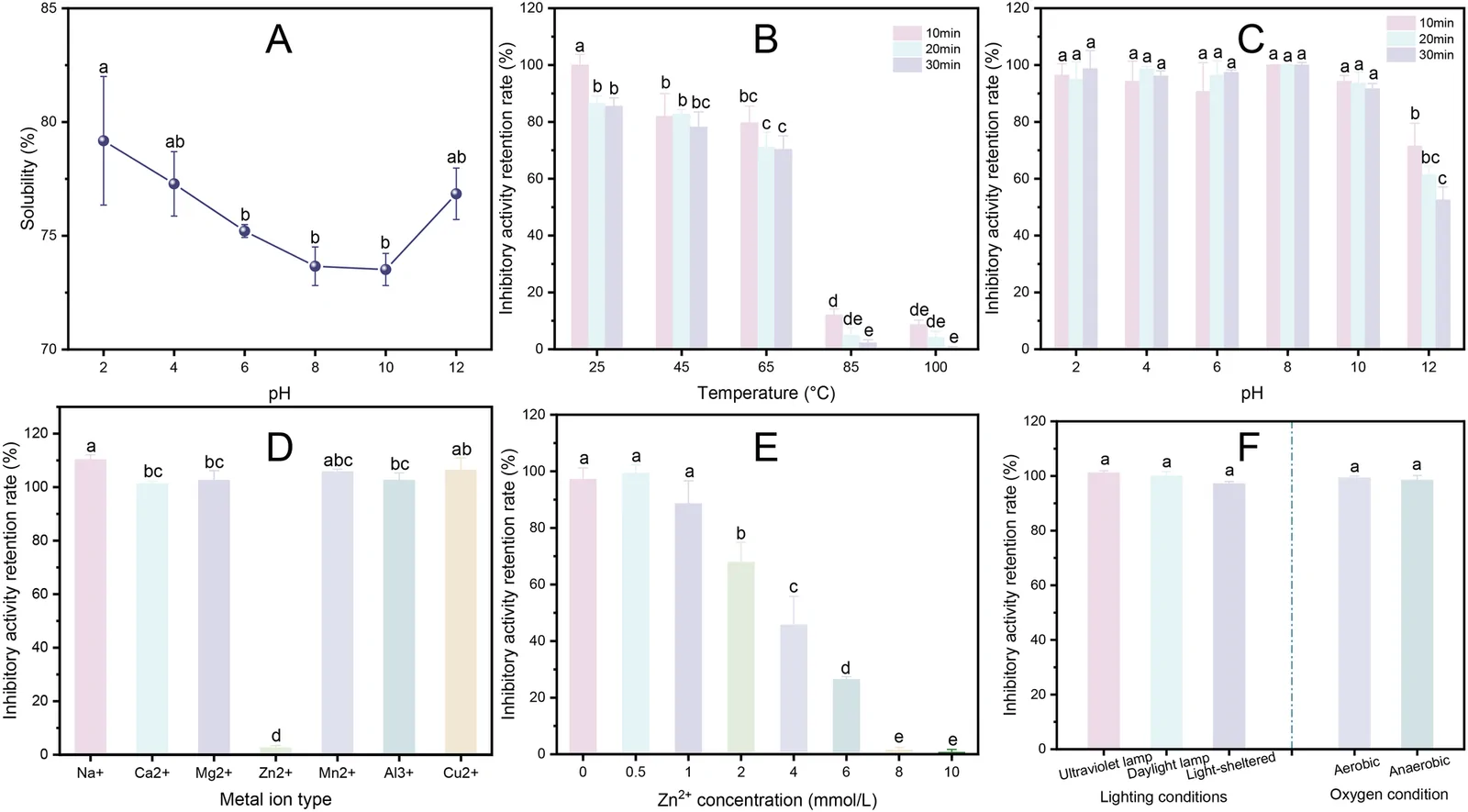
Solubility of PTI at different pH and the stability of its inhibitory activity under various conditions. **A** Solubility of PTI at different pH. **B** Effect of temperature on inhibitory activity retention. **C** Effect of pH on inhibitory activity retention. **D** Effect of metal ions on inhibitory activity retention. **E** Effect of Zn^2+^ concentration on inhibitory activity retention. **F** Effect of light and oxygen conditions on inhibitory activity retention. Different letters indicate significant differences (*p* < 0.05).

By analyzing Fig. 5A, it can be seen that the solubility of PTI at room temperature is relatively high (roughly 73–80%) over a wide range of pH, which is similar to the findings of Ralet et al.^16^. The solubility of PTI showed a decrease and then an increase with increasing pH. The optimal pH for PTI solubility was pH 2.0 (~79%), followed by pH 4.0 (~77%) (Fig. 5A). At these acidic pH values, PTI carries a net positive charge due to protonation of amino acid residues, generating electrostatic repulsion that prevents aggregation. Solubility decreased at pH 8.0–10.0, which is closer to the isoelectric point (pI 5.1–9.0) of PTI, where reduced net charge promotes protein precipitation^41^. It has been reported that when the pH is at the isoelectric point of proteins, the number of positive and negative ions dissociated from the polar groups in the protein molecules reaches an equilibrium, resulting in a net charge of 0 for the protein molecules as a whole. Meanwhile, due to the lack of mutual repulsion between the same charges, the interaction force between the protein molecules is weakened, making it easier for the protein particles to collide, aggregate, and ultimately form a precipitate^42^.

### Stability of PTI

Heat treatment is an indispensable process in the field of food processing. When food products are subjected to heat treatment at a temperature interval of 60 °C to 90 °C, the protein components therein may be denatured and aggregated. The results of the residual inhibitory activity of PTI after different gradients of temperature treatments are illustrated in Fig. 5B, where a decreasing trend in the retention of inhibitory activity of PTI with a gradual increase in temperature can be observed. In the range of temperature below 65 °C, the effect of temperature on the inhibitory activity of PTI was relatively limited, which means that PTI can maintain relatively stable activity in this temperature interval, and thus it is suitable for treatment processes such as pasteurization. However, when the temperature further increased to 75 °C, the inhibitory activity of PTI showed a significant decrease, and the retention rate of its inhibitory activity plummeted to 26.32% at a treatment time of 10 min, which indicated that the heating temperature of 75 °C had a significant inactivation effect on PTI. After further treatment at 100 °C for 30 min, only 0.38% of the inhibitory activity of PTI remained. Besides, prolonging the heating time, the retention of inhibitory activity of PTI exhibited a decreasing trend at all temperatures, which was similar to the findings in other studies^13^.

In Fig. 5C, there was no significant change in the retention rate of PTI inhibitory activity in the range of pH 2~10, which were all above 85%, and there was also no significant change in the retention time of 10~30 min (*p* > 0.05), which indicated that PTI had better pH stability and a wider range of applications. However, when the pH reached 12, the retention of PTI inhibitory activity decreased significantly, and with the extension of time, the retention of PTI inhibitory activity decreased further. This might be due to the fact that some of the active peptide fragments were further decomposed and transformed into inactive fragments under the combined effect of strong alkalinity and heating, which was similarly concluded in other studies^13^. Overall, PTI not only demonstrated a certain heat resistance but also maintained a certain adaptability over a wide pH range, which further enriches and extends the current understanding of the temperature and pH stability studies of PTI.

The results of retention of inhibitory activity of PTI after treatment with different metal ions at a concentration of 10 mM are presented in Fig. 5D. From the figure, it can be seen that the addition of appropriate amounts of Na^+^ and Cu^2+^ helped to improve the inhibitory activity of PTI, and the retention of inhibitory activity of PTI reached 110.25% with the addition of Na^+^, which increased the inhibitory activity by 10.25%, whereas the addition of Ca^2+^, Mg^2+^, Mn^2+^, and Al^3+^ did not have any significant effect on the retention of inhibitory activity of PTI (*p* > 0.05). However, different from the other metal ions, the addition of Zn^2+^ caused the inhibitory activity of PTI to plummet with only 2.56% retention of activity, which was considered to be possibly due to the fact that the addition of Zn^2+^ precipitates the PTI from the solution^43^. Therefore, subsequent experiments with different concentrations of Zn^2+^ were set up to investigate the effect of its concentration on the retention of PTI inhibitory activity.

Figure 5E plots the results of retention of PTI inhibitory activity after treatment with different concentrations of Zn^2+^. As can be seen from the figure, when the concentration of Zn^2+^ was very low (0.5 mM), the inhibitory activity of Zn^2+^ on PTI was slightly increased, but the effect was not significant (*p* > 0.05). Whereas, when the concentration of Zn^2+^ reached 2 mM, there was a significant decrease in the retention of PTI inhibitory activity compared to the blank (*p* < 0.05). When the concentration of Zn^2+^ was in the range of 1 to 8 mM, the retention of PTI inhibitory activity was decreasing with the increase in concentration and the effect was significant (*p* < 0.05). When the concentration of Zn^2+^ reached 8 mM, the retention rate of PTI inhibitory activity was only 2.14%, and there was no longer any change when the concentration was increased to 10 mM, which indicated that the 8 mM concentration of Zn^2+^ could completely inhibit the inhibitory activity of PTI, and the effect of the reduction of the PTI inhibitory activity would remain basically unchanged even if the concentration of Zn^2+^ was increased subsequently. Additionally, it was found that the addition of Zn^2+^ led to the precipitation of PTI from the PTI solution, and the amount of PTI precipitated increased with the increase of Zn^2+^ concentration. This means that the principle of PTI inhibition by Zn^2+^ is that the addition of Zn^2+^ causes the precipitation of PTI from the PTI solution, which leads to a decrease in the concentration of PTI in the solution, resulting in a decrease in the retention of PTI inhibitory activity^44^. Moreover, it was proved that the process was reversible, and after mixing the reaction solution with precipitation, the same inhibitory activity as that of the blank group was retained in the solution.

In Fig. 5F, there was no significant change (*p* > 0.05) in the retention of inhibitory activity of PTI under different light conditions (UV lamp, daylight lamp, and light-sheltered), which indicates the feasibility of employing UV sterilization as a treatment for PTI^45^. Similarly, there was no significant difference (*p* > 0.05) in the retention of inhibitory activity of PTI under aerobic and anaerobic conditions, suggesting that exposure to air would not decrease the inhibitory activity of PTI during short-term storage.

**Fig. 6:**
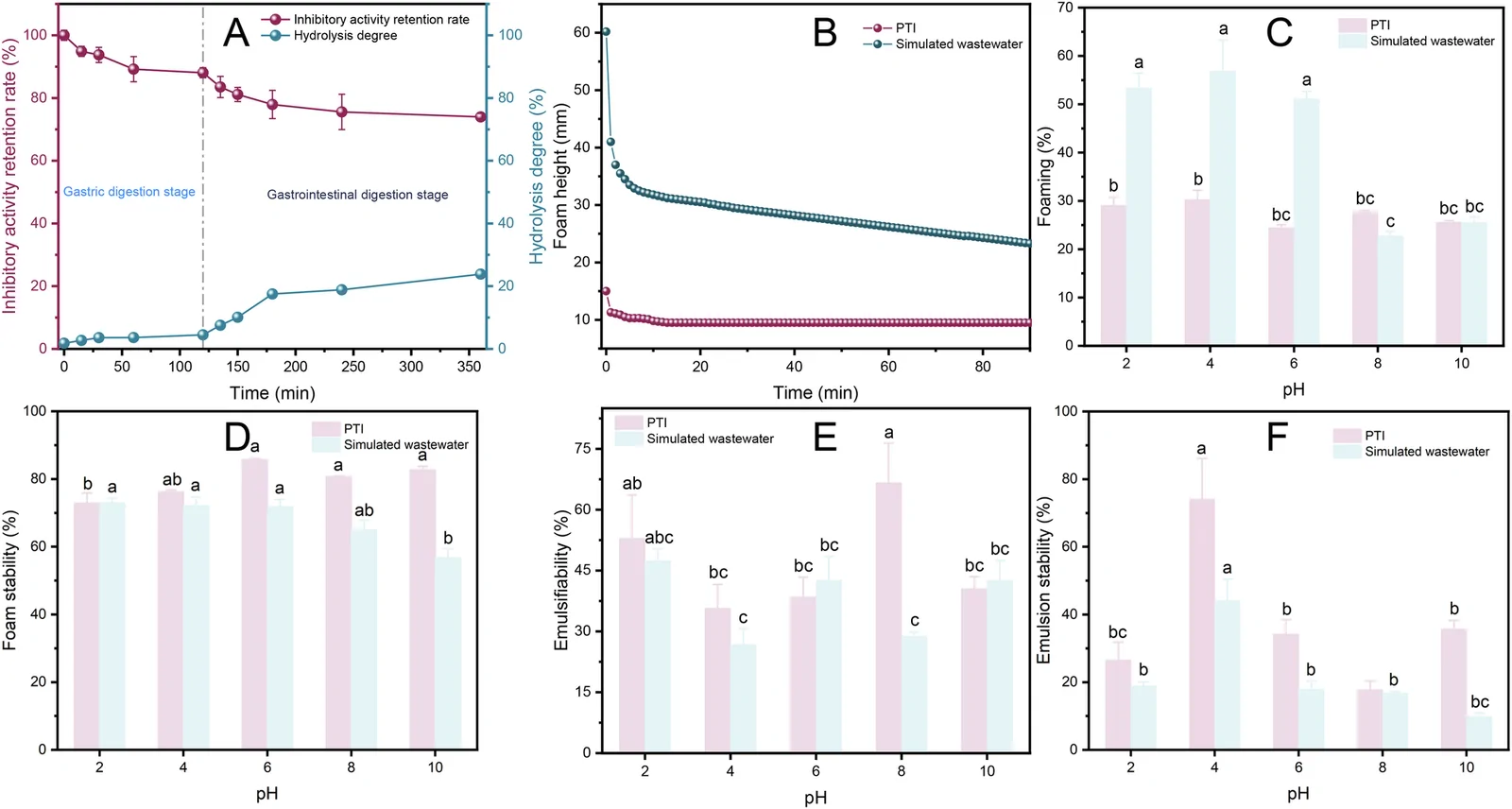
In vitro digestion stability, foaming properties, and emulsifying properties of PTI and potato simulated wastewater. (**A**) In vitro digestion stability and degree of hydrolysis of PTI. (**B**) Foam height variation over time for PTI and potato simulated wastewater. (**C**) Foaming capacity at different pH. (**D**) Foam stability at different pH. (**E**) Emulsifying activity at different pH. (**F**) Emulsion stability at different pH. Different letters indicate significant differences (*p* < 0.05).

The retention of trypsin inhibitory activity as well as the degree of protein hydrolysis of PTI during simulated digestion of PTI in *vitro* are shown in Fig. 6A. The left part of the dashed line is simulated gastric digestion, and the right part is simulated intestinal digestion. It can be found that the simulated gastric digestion exhibited a rapid decrease in PTI inhibitory activity at the beginning of the process (0~15 min), and this significant change is most likely attributed to the very low pH environment in the gastric juice (pH 1.2), which adversely affects some of the activities of PT^13^. The rate of decrease in PTI inhibitor activity gradually slowed down as the digestion process progressed and finally reached 88.07% of the initial activity. Similar to the trend of simulated gastric digestion, during simulated intestinal digestion, starting from 120 min, the rate of decrease of PTI inhibitory activity gradually slowed down with time and finally reached 73.98% of the initial simulated digestion.

In Fig. 6A, the initial protein hydrolysis degree of PTI was 1.79%, and when exposed to the gastric fluid environment for digestion, the protein hydrolysis degree value of PTI rendered a small increase to 4.48%. During the intestinal digestion phase, its hydrolysis degree showed a more significant increase, finally reaching about 23.83% at the end of the intestinal fluid digestion (360 min). Compared with the rate of loss of inhibitory activity during in vitro digestion, the degree of protein hydrolysis of PTI was relatively low. In summary, PTI exhibits some resistance to gastrointestinal digestion, a property that may be attributed to the antiparallel β-fold structure enriched in its structure^13^. In fact, Vagadia et al.^46^ explained this similar result by the fact that soybean Kunitz-type trypsin inhibitors, which are mainly characterized by an antiparallel β-folding structure, are able to effectively resist the enzymatic catabolism in gastric juice.

### Foaming properties and foam stability of PTI

The purpose of characterizing PTI’s emulsifying and foaming properties is not to directly correlate them with its trypsin inhibitor function, but rather to comprehensively evaluate its potential as a multifunctional food ingredient. While PTI’s core value lies in its biological activity, its essential processing functionalities as a protein are crucial for successful application in complex food systems such as beverages and dairy products. As can be seen from Fig. 6B, under the condition of no pH adjustment, the foaming property of PTI was lower than that of potato simulated wastewater, which may be due to the fact that potato wastewater contains some glycoproteins, which makes the foaming property higher^3^. The foam height of the PTI group reached equilibrium in about 10 min, while that of the potato simulated wastewater group decreased rapidly in 10 min and slowly from 10 to 90 min.

The effect of different pH conditions on the foaming properties of PTI in Fig. 6C did not change significantly (*p* > 0.05), which may be due to the fact that the solubility of PTI is relatively high over a wide range, resulting in the absence of protein precipitation in the solution when the pH is changed. The foaming property of the simulated wastewater was higher in the acidic environment, and there was a significant decrease at pH 8 and 10, which is consistent with the results in Fig. 5A, owing to the fact that the isoelectric point of proteins in the potato wastewater is around pH 8–10, which causes some proteins to be precipitated at pH 8 and 10 and leads to a decrease in the foaming property^41^.

In Fig. 6D, the foam stability of PTI gradually increased between pH 2 and 6, reaching a peak (over 80%) before decreasing slightly at pH 8 and 10, but generally remaining at a high level. In contrast, the foam stability of the simulated wastewater varied very little between pH 2 and 6, but decreased more significantly at pH 8 and 10, especially at pH 10, which was attributed to be closely related to the fact that the foaming properties of the simulated wastewater, as described above, decreased significantly at pH 8 and 10, whereas the foaming properties of the PTI were almost unaffected at pH 8 and 10^41^.

### Emulsifiability and emulsion stability of PTI

Emulsifiability and emulsion stability are two common indicators for evaluating the emulsifying properties of proteins. Emulsifiability mainly reflects the degree of protein involvement in the emulsion formation process, which measures the ability of proteins to promote the formation of emulsions; while emulsion stability further considers the efficacy of proteins in maintaining the stability of emulsions, which is specifically expressed as whether the emulsions are able to avoid the phenomena of delamination and flocculation within a specific time period^47^. As can be seen from the data presented in Fig. 6E, the emulsification properties of both PTI and simulated wastewater protein freeze-dried powders presented a multiple undulating pattern of change between emulsification and pH, which first declined, then increased, then declined and finally increased again. However, when comparing the emulsification properties of the two groups of samples, it can be observed that PTI exhibited overall good emulsification properties to the simulated wastewater freeze-dried powder. In particular, the emulsification performance of PTI reached its optimum at pH 8, with an emulsification of 66.68%. The emulsifiability of proteins is generally closely related to their conformation and charge state in solution. Under different pH environments, the charge state of proteins changes, which affects their interactions with other molecules and their arrangement at the water-oil interface^47^. For PTI, it may be in a more stable charge state when the pH is 8, which is favorable for the formation of a close and orderly arrangement at the oil-water interface, thus enhancing the emulsification performance. Moreover, PTI, as a protease inhibitor, has a specific amino acid sequence and spatial structure, and these structural features enable PTI to better perform its emulsifying effect under specific pH conditions^48^. In particular, in a near-neutral or weakly alkaline environment (e.g., pH 8), the spatial structure of PTI may be more stable, which is conducive to its interaction with the surface of the oil droplets, thus improving the emulsification effect^49^.

Figure 6F depicts the emulsification stability of simulated wastewater and PTI under different pH conditions, which showed a linear increase and then decreased with the increase of pH value. The emulsification stability of PTI was higher than that of simulated wastewater, and the emulsification stability of both PTI and simulated wastewater reached the maximum (74.06% and 44.05%) at pH 4, which may be due to the fact that its molecular structure is most conducive to the stabilization of the emulsification system at this pH value^13,50^. In contrast, the emulsion stability of simulated wastewater was less sensitive to pH changes and remained low and stable, which may be due to its complex composition, which contains a variety of factors that are not conducive to the stability of emulsification.

The emulsifiability and emulsion stability of PTI were evaluated at different pH values (2, 4, 6, 8, 10) and compared with potato simulated wastewater freeze-dried powder. As shown in Fig. 6E, the emulsifiability of PTI reached its maximum at pH 8 (66.68%), while the emulsion stability was highest at pH 4 (74.06%) (Fig. 6F). PTI consistently outperformed the crude wastewater extract across all pH conditions, indicating that purification enhances emulsifying performance.

Several considerations should be noted regarding the emulsifying properties of PTI. First, the optimal emulsifiability at pH 8 can be attributed to an ideal balance between protein solubility (Fig. 6A) and surface hydrophobicity (Fig. 4A), which promotes rapid adsorption to the oil-water interface. Second, the highest emulsion stability at pH 4, near the isoelectric point of PTI, suggests that reduced electrostatic repulsion allows closer protein packing at the interface, forming a more cohesive and stable film. Third, the superior performance of purified PTI compared to crude wastewater extract reflects the removal of interfering components (sugars, glycoproteins) that compete for interface space or disrupt film formation. These considerations highlight PTI’s potential as a dual-functional emulsifier in food applications.

The experimental results of this work were interrelated, collectively forming a complete chain of evidence from method screening to structural characterization and then to functional validation. This systematically supported the core conclusion that acid precipitation-salting out is the optimal method for preparing highly functional PTI.

First, there was a clear causal relationship between the comparison of extraction methods and the structural characterization of PTI. The acid precipitation-salting-out method yielded PTI with the highest purity among the three methods and best activity because its stepwise precipitation strategy efficiently removed impurities such as glycoproteins (Fig. S1). The resulting PTI exhibited enhanced structural stability, as evidenced by higher trans-conformation absorption peaks in its FTIR spectrum (Fig. 2A), richer β-sheet structures (Table 1), narrower particle size distribution, and higher absolute zeta potential values (Fig. 4A, B). These structural features directly explained their superior performance in subsequent stability experiments. In contrast, while direct adsorption achieved high yields, the resin’s non-specific adsorption led to co-purification of sugars and pigments. This not only reduced product purity (Table 2), but its harsh physicochemical environment may have disrupted PTI’s native conformation, resulting in decreased β-sheet proportion and increased β-turn proportion (Table 1), which may represent the structural basis for its limited functionality. The metal ion precipitation method was excluded due to unacceptable safety risks (extremely high zinc residues, Table S2), underscoring that product safety remains the decisive factor in method selection when pursuing efficiency.

Secondly, the exceptional structural characteristics of PTI directly determine its outstanding stability and functional properties. The abundant β-sheet structures in PTI, particularly antiparallel β-sheets, formed the key molecular basis for its tolerance across a broad pH range (Fig. 5C) and resistance to gastrointestinal digestion (Fig. 6A). This aligns with literature-reported findings that such structures effectively resisted protease hydrolysis. Its small particle size and high surface charge (zeta potential) confer excellent physical stability in solution systems, which is crucial for its emulsifying and foaming properties. Notably, although PTI obtained *via* direct adsorption exhibits higher surface hydrophobicity (Fig. 3A), its inferior emulsifying and foaming properties (Fig. 6E, F) indicated that protein functionality depends not only on the extent of hydrophobic group exposure but also on the integrity of its overall structure and its ability to arrange in an ordered manner at the interface. PTI obtained via acid precipitation-salting out likely retains a more natural, flexible conformation, enabling it to unfold and rearrange more effectively at oil-water or gas-water interfaces to form stable interfacial films.

Finally, all these findings collectively supported one conclusion: PTI extracted from potato wastewater via the acid precipitation-salting out method is a high-value ingredient that combines high purity, high activity, outstanding stability (thermal, pH, digestion), and excellent processing functionality (emulsification, foaming). This work not only systematically identified the optimal extraction process through comparative analysis but, more importantly, elucidated the intrinsic logic by linking extraction method-structural characteristics-functional properties, which provided comprehensive theoretical foundations and practical guidance for utilizing PTI as a sustainable and multifunctional food ingredient in complex food systems (e.g., beverages, dairy products, meat alternatives). This approach not only achieves waste-to-value conversion but also pioneers new raw material pathways for developing clean-label functional foods.

## Discussion

This study systematically compared three extraction strategies—acid precipitation-salting out, direct resin adsorption, and zinc ion precipitation—for PTI recovery from potato processing wastewater under identical conditions using the same raw material and analytical platform. Among the three methods, acid precipitation-salting out emerged as the most effective approach, yielding PTI with the highest purity (63.62% protein) and superior specific activity (533.06 TIU/mg). The stepwise precipitation strategy efficiently removed heteroproteins, particularly glycoproteins, resulting in a product with enhanced structural integrity, as evidenced by a higher β-sheet content (48.09%), narrower particle size distribution, and elevated absolute zeta potential. These structural advantages translated directly into superior functional performance, including favorable emulsifying capacity (66.68% at pH 8), foam stability exceeding 80%, and broad pH stability. In contrast, direct resin adsorption achieved a higher yield (1287 mg/kg) but significantly lower purity (40.69%) and purification factor (1.55). The resin’s non-specific adsorption led to co-purification of sugars and pigments, and the harsh physicochemical environment during adsorption and desorption likely induced partial unfolding of PTI, as reflected by its higher β-turn content (40.38%), lower β-sheet content (22.00%), and increased surface hydrophobicity. Although higher surface hydrophobicity is generally expected to enhance interfacial activity, the direct adsorption product exhibited inferior emulsifying and foaming properties, suggesting that protein functionality depends not merely on hydrophobic group exposure but also on the integrity of the overall conformation and the protein’s ability to arrange in an ordered manner at interfaces^11,13,14,16^. Zinc ion precipitation, while demonstrating promising protein recovery efficiency and achieving 40.16% inhibitory activity recovery at 6 mM Zn^2+^, was excluded from further characterization due to unacceptably high zinc residues in the final product (41,599.05 mg/kg in freeze-dried powder, Table S2), far exceeding safety limits for food ingredients. This negative result is itself valuable for the field, as it provides the first quantitative safety assessment of metal ion precipitation for PTI recovery and serves as a warning against adopting this approach without rigorous heavy metal removal steps. The mechanism of Zn^2+^-induced protein precipitation observed here is consistent with previous reports that metal ions can form ternary complexes with polyphenolic compounds and proteins, leading to co-precipitation^23,34^.

Previous studies have successfully extracted PTI using acid precipitation-salting out but focused on single-method optimization and activity-only characterization^11,13–16^. The present study differs by providing: (1) the first parallel comparison of three extraction strategies for PTI under identical conditions; (2) the first quantitative safety assessment of metal ion precipitation in terms of Zn^2+^ residues; and (3) the first complete structure-techno-function relationship linking extraction method to detailed structural characteristics (FTIR-derived secondary structure, surface hydrophobicity, particle size distribution, zeta potential, intrinsic fluorescence) and further to emulsifying and foaming properties. Furthermore, this study provides the first systematic evaluation of PTI stability under previously unreported conditions, including exposure to seven different metal ions (Na^+^, Cu^2+^, Ca^2+^, Mg^2+^, Zn^2+^, Mn^2+^, Al^3+^), various light sources (UV lamp, daylight lamp, dark condition), and both aerobic and anaerobic environments. The finding that Zn^2+^ at concentrations as low as 0.5 mM can precipitate PTI from solution, with near-complete activity loss at 8 mM, provides mechanistic insight into the metal ion sensitivity of this protease inhibitor and has practical implications for food formulation where metal ions may be present^43,44^. Additionally, the resistance of PTI to simulated gastrointestinal digestion (73.98% activity retention) observed in this study is consistent with previous findings and can be attributed to the antiparallel β-sheet structure enriched in Kunitz-type trypsin inhibitors, which effectively resists enzymatic degradation in gastric fluid^13,46^. These contributions collectively distinguish the present work from previous single-method studies and provide comprehensive theoretical foundations for utilizing PTI as a sustainable and multifunctional food ingredient.

Several limitations of this study should be acknowledged. First, the simulated potato wastewater, while standardized and reproducible, may not fully capture the compositional variability of real industrial potato processing wastewater, which can vary with potato cultivar, season, and processing conditions^2,3,10^. Second, all extractions were performed at laboratory scale (200 mL batches); pilot-scale validation using real industrial wastewater is necessary to assess scalability, particularly regarding large-volume pH control, efficient precipitate separation, salt recovery, and batch-to-batch consistency. Third, techno-functional properties (emulsification, foaming) were evaluated in simple model systems (1% protein solutions with vegetable oil or air); performance in complex food matrices such as multi-ingredient beverages, high-sugar or high-salt systems remains to be investigated^3,12^. Fourth, while the Zn^2+^ precipitation method was excluded due to heavy metal residue concerns, chelation or extensive washing steps that might reduce zinc residues to acceptable levels were not explored, which could be a direction for future investigation if the high precipitation efficiency of this method is to be harnessed. Fifth, the long-term storage stability of the lyophilized PTI powder (e.g., 6–12 months at different temperatures) was not evaluated. Future studies should address these limitations before industrial application.

The acid precipitation–salting out method is potentially scalable to an industrial level, as both unit operations are standard and low-cost in the food industry. Looking forward, further innovations are worth exploring. Future work could investigate green extraction technologies such as ultrasound-assisted or microwave-assisted extraction to reduce processing time and chemical consumption, membrane-integrated continuous processing systems to improve scalability, enzymatic pretreatment strategies to selectively remove non-PTI proteins before acid precipitation, and a comprehensive techno-economic analysis to guide industrial implementation of the optimal method identified in this study^3,51^. Such advances would further support the sustainable valorization of potato processing waste into high-value functional ingredients for the food industry.

In summary, this work not only establishes acid precipitation-salting out as a scalable, safe, and efficient strategy for high-quality PTI extraction but also provides the first complete chain of evidence linking the extraction method to structural characteristics and techno-functional performance. The dual functionality of PTI—combining potent trypsin inhibitory activity with favorable emulsifying and foaming properties—positions it as a promising natural ingredient for innovative food applications, supporting the circular bioeconomy through waste-to-value conversion^2,3,11^.

## Methods

### Material and chemicals

Edible-grade potatoes were sourced from Guangzhou Huidong Agricultural Products Company in Guangzhou, China. Commercial enzyme (trypsin) was purchased from Novozymes (China) Biotechnology Ltd (Tianjin, China). The different resins (D-101 resin, HPD82 resin, 30-60mesh resin, ADS-600 resin, 732 Anion resin (Na), AB-8 resin, LX-8 resin, HPD-400 resin, XDA-1 resin, LX-38 resin, HPD-600 resin, XAD-16 resin, LXTE-600 resin, and LXTE-603 resin) and hydrated silica were purchased from Shanghai MackLin Biochemical Technology Co., Ltd (Shanghai, China). All remaining reagents, of analytical grade, were obtained from Wuhan Seville Biological Technology Co., Ltd, located in Wuhan, China. Purified water, with a resistivity of 18.2 MΩ, was generated using a Millipore Mill-Q Ultrapure Water System, manufactured in Billerica, USA.

### Preparation of potato simulated processing wastewater

**Fig. 7:**
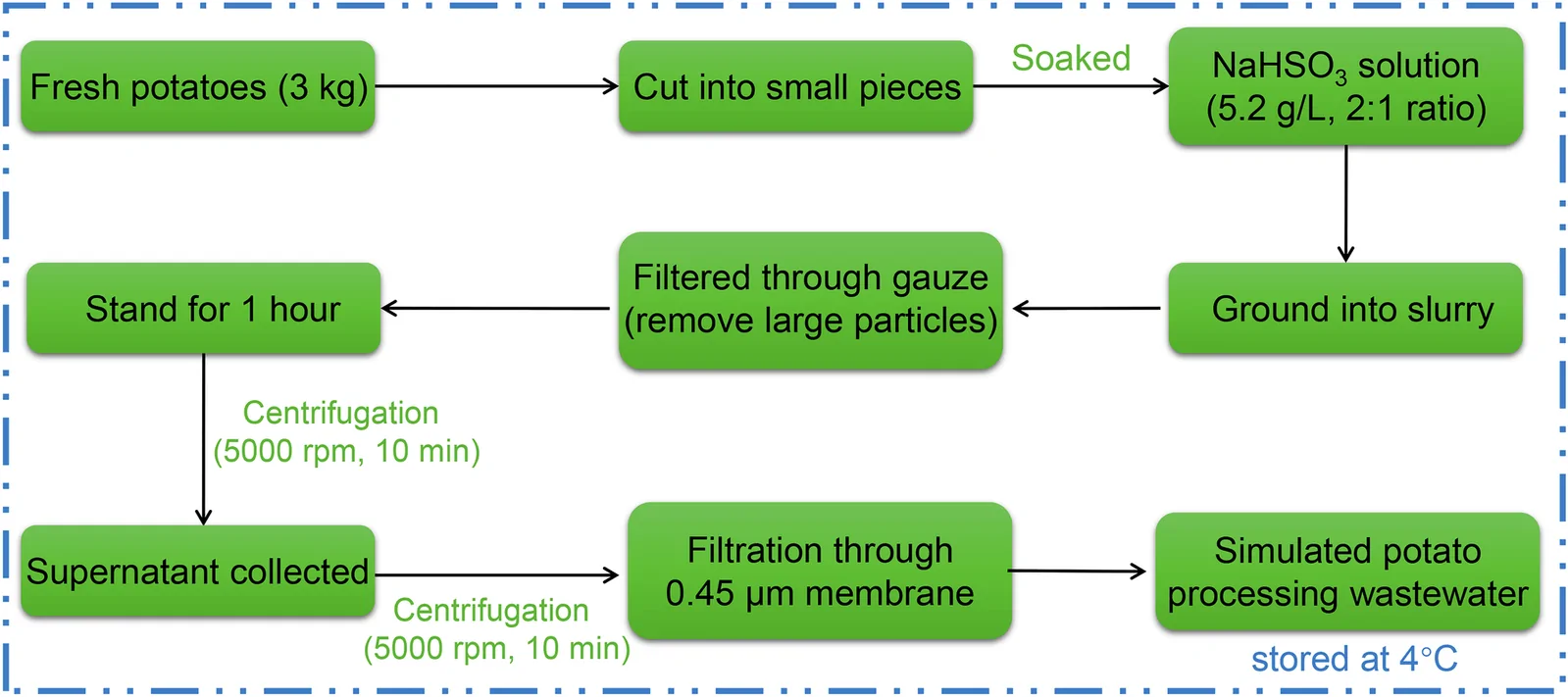
Schematic diagram of the preparation process. The diagram illustrates the sequential steps from potato wastewater to the final PTI product via the two preparation methods (acid precipitation combined with salting out, and direct adsorption).

The preparation of potato simulated processing wastewater was improved on the scheme of Li et al.^13^. Potatoes of 3 kg were cleaned and subsequently cut into small pieces. They were then soaked in a sodium bisulphite solution with a concentration of 5.2 g/L, using a ratio of 2:1 potato pieces to solution. The mixture was processed into a slurry using a meat grinder (model JR16-300, manufactured by Zhejiang Shaoxing Supor Household Products Co., Ltd). Initially, the slurry was filtered through gauze to remove larger solid particles. The filtered mixture was allowed to stand for 1 h before being centrifuged at 5000 rpm for 10 min using a centrifuge (model GL-21M, manufactured by Changsha Xiangyi Centrifuge Co., Ltd). The supernatant portion was collected and further pumped through a 0.45 μm microporous filter membrane (Millipore Merck & Co., Inc.) to remove impurities, and the resulting filtrate was the potato starch wastewater, which was subsequently stored in a refrigerator (BCD-607WKPZM, Midea Group Corporation) at 4 °C for subsequent experiments (Fig. 7).

### Determination of protein and total sugar content of potato simulated processing wastewater

The soluble protein content of potato wastewater was determined using a BCA kit, and the total nitrogen of the final isolated product PTI was determined using Kjeldahl nitrogen determination^52^. The phenol-sulfuric acid method was used to determine the total sugar content^53^. For measurement, a 1 mL aliquot of the sample (with 1 mL of distilled water as the blank control) was pipetted into a 10 mL stoppered test tube. To this, 1 mL of 5% phenol solution and 5 mL of concentrated sulfuric acid were promptly dispensed. The contents were thoroughly mixed and allowed to stand for 1 min. Following this, the mixture was subjected to heating in a water bath (model Bit1000, manufactured by Hangzhou Ruicheng Instrument Co., Ltd) at a temperature of 30 °C for 20 min, and then allowed to cool down to room temperature. The absorbance of the mixture was then determined using a spectrophotometer (model UV8000, manufactured by Shanghai Yuan Analysis Instrument Co., Ltd) at a wavelength of 490 nm, denoted as OD_490_. Formula (1) of the standard curve was obtained by preparing a series of glucose solutions of different concentrations (0, 0.02 0.04, 0.06, 0.08, and 0.1 mg/mL) with an *R*² of 0.9916, and measuring their absorbance values under the same conditions, so that the total sugar content in the sample could be calculated from the measured OD_490_ of the mixture.

1 $${\rm{Y}}=5.45\,{\rm{X}}+0.0626$$

Where *Y* represents the OD_490_ value and *X* represents the glucose solution.

### Determination of PTI activity

The quantification of PTI activity primarily depends on its capacity to inhibit trypsin activity. Specifically, one unit of PTI activity, known as TIU, is defined as the ability to inhibit one unit of trypsin activity. The determination of PTI activity was carried out with reference to the traditional Acetyltoluidine hydrochloride (BAPNA) method utilized by Gujjarlapudi et al.^54^. For each sample, three replicates were set up, and the samples were diluted with Tris-HCl buffer (50 mM) to an appropriate concentration, ensuring an inhibition range between 40% and 60% prior to conducting the assay. Tris-HCl buffer (50 mM, pH 8.2) was selected for PTI activity assays and stability studies because (i) PTI exhibits maximal inhibitory activity at pH 8.0–8.5^13,15^, (ii) the BAPNA assay is standardized at this pH, and (iii) Tris-HCl does not chelate metal ions, which is critical for the metal ion stability experiments.

The specific procedure was outlined as follows: Both the sample group and its corresponding blank control group, as well as the standard group and its corresponding blank control group, were established. In the sample group, 2 mL of the diluted sample solution was pipetted into a test tube. Subsequently, 5 mL of a 0.04% BAPNA solution was added and mixed homogeneously in a water bath maintained at 37 °C for 10 min. Afterward, 2 mL of a 0.01% trypsin solution, also mixed homogeneously in a 37 °C water bath, was rapidly introduced to initiate a precise 10 min reaction. The reaction was then terminated by the addition of 1 mL of a 30% glacial acetic acid solution. The absorbance value of the reaction mixture at 410 nm (OD_410_) was measured and recorded as A_sample_. For the corresponding sample blank control, ultrapure water was used in place of the 0.01% trypsin solution to determine PTI activity.

In the standard group, a test tube was filled with 2 mL of Tris-HCl buffer. Then 5 mL of a 0.04% BAPNA solution was added and thoroughly mixed in a water bath maintained at 37 °C for 10 min. Subsequently, 2 mL of a 0.01% trypsin solution, also mixed homogeneously, was swiftly introduced into the test tube to initiate a precise reaction at 37 °C for another 10 min. The reaction was promptly terminated by the addition of 1 mL of a 30% glacial acetic acid solution. The OD410 was then measured and recorded as *A*_standard_. For the corresponding standard blank control, ultrapure water was substituted for the 0.01% trypsin solution in the standard set to determine PTI activity. The TIU value can be computed using Formula (2) as follows:

2 $$\mathrm{TIU}=\frac{[\left({\mathrm{A}}_{\mathrm{standar}}-{\mathrm{A}}_{\mathrm{ck}2}\right)-\left({\mathrm{A}}_{\mathrm{sample}}-{\mathrm{A}}_{\mathrm{ck}1}\right)]}{\left({\mathrm{A}}_{\mathrm{standar}}-{\mathrm{A}}_{\mathrm{ck}2}\right)\times \mathrm{W}}\times \mathrm{D}$$

Where *A*_ck1_ represents the OD410 of the sample blank control, *A*_ck2_ represents the OD410 of the standard blank control, D represents the dilution factor, and *W* represents the weight of the test sample (mg).

### Sodium dodecyl sulfate polyacrylamide gel electrophoresis (SDS-PAGE)

The purity of PTI was characterized by discontinuous SDS-PAGE electrophoresis, referring to the method of Jiang et al.^55^ with minor modifications. The supernatant was combined with an equal volume of SDS-PAGE sample buffer (P0015B, supplied by Beyotime Biotechnology Co., Ltd) and subjected to boiling at 100 °C for a duration of 5 min to denature the proteins. Following boiling, 10 µL of each processed sample were loaded into an electrophoresis unit (Mini-Sub Cell, manufactured by Bio-Rad Inc.), which contained a 120 g/L electrophoresis gel overlaid with a 50 g/L stacking gel. During the electrophoresis process, the voltage was adjusted to 80 V for the stacking gel and increased to 120 V for the electrophoresis gel. Upon completion of electrophoresis, the gels were stained for 2 h using Thomas Brilliant Blue Ultra-Fast Staining Solution (P0017F, Beyotime Biotechnology Co., Ltd). Subsequently, the stained gel was destained with distilled water for an additional 2 h. Protein markers with molecular weights ranging from 10 to 180 kDa (Thermo Scientific™) were utilized to estimate the molecular weights of the proteins.

### Extraction of PTI

With reference to the method of Li et al., the trypsin inhibitor in potato wastewater was extracted and separated by acid precipitation combined with salting out^13^. For acid precipitation, 20 mL of potato wastewater was taken, and the pH was adjusted to 2, 2.5, 3, 3.5, 4, 4.5, and 5 with 1 M HCl, which was recorded as S2, S2.5, S3, S3.5, S4, S4.5, and S5, respectively. Subsequently, the solution was allowed to stand at room temperature for 20 min, and after centrifugation (25 °C, 5000 rpm) for 10 min, the supernatants were taken to determine the protein content, inhibitory activity, and electrophoretic analysis, respectively.

In the salting out process, 200 mL of potato wastewater was first acid precipitated and decontaminated, and the supernatant was taken and 20, 25, 30, 35, 40% saturated NaCl, and 40, 50, 60, 70% saturated (NH_4_)_2_SO_4_ were added to it, which were noted as Y1, Y2, Y3, Y4, Y5, Y6, Y7, Y8, and Y9, respectively.

After the complete dissolution of the salt was observed, stirring continued for about 30 min to ensure that the salt was uniformly and fully dissolved. The treated solution was placed in a refrigerator at 4 °C overnight, and the precipitate was collected by centrifugation at 8000 rpm for 20 min. The precipitate was then re-dissolved in the same volume of deionized water, followed by the determination of protein content and inhibitory activity of the solutions before and after treatment to obtain the protein recovery rate and inhibitory activity recovery rate of the inhibitor before and after treatment.

The direct adsorption method is based on the method of Zeng et al. with corresponding modifications^56^. In the pre-treatment of the resin, the appropriate amount of resin was weighed in a funnel and rinsed with distilled water and then soaked in 95% ethanol for 24 h after filtering out the water, followed by vacuum filtration and rinsing with distilled water until the resin was free of alcohol odor.

In the adsorption treatment, different material-liquid ratios (1:10, 1:5 and 2:5) were set, and different types of adsorption material and potato wastewater were weighed and added into 50 mL triangular flasks and shaken for 6 h at 25 °C and 200 rpm. At the end of the adsorption, the resin and adsorbent were filtered and separated, and the protein content and inhibitory activity in the adsorbent were determined to calculate the protein adsorption rate and inhibitory activity adsorption rate, while the resin continued to be pumped and filtered until it was free of water.

For desorption, 0.1 g of adsorbed resin and 5 mL of desorbent were placed in a 50 mL triangular flask and shaken at 25 °C and 200 rpm for 12 h, respectively. After desorption, the resin and desorbent were separated by filtration to determine the protein content and inhibitory activity in the desorbent. The samples of ethanol and methanol groups were freeze-dried after rotary evaporation (60 °C, 45 bar), and the samples of NaCl and NaOH groups were freeze-dried (model YTLG-10A, Shanghai YETO Technology Co., Ltd) after ultrafiltration using 10 kDa ultrafiltration membrane (Millipore Merck & Co., Inc.).

The zinc ion precipitation method is based on the method of Zeng et al. with minor modifications^19^. At room temperature, different metal ions solutions (Ca^2+^, Mg^2+^, Zn^2+^) were added to the potato wastewater so that the final concentrations of metal ions were 4, 6, 8 and 10 mM. The sample solution was allowed to stand for 2 h and centrifuged at 5000 rpm for 10 min, and the supernatant was extracted to determine the protein content and the PTI inhibitory activity, to calculate the recoveries of the inhibitors before and after treatment.

Moreover, an appropriate amount of potato wastewater was taken to adjust the pH to 5.3, 5.7, 6.0, 6.5 and 7, respectively, followed by the addition of metal ion solution to make the final concentration of Zn^2+^ 1, 2, 4, 6 and 8 mM, respectively. After the sample solutions were allowed to stand for 2 h and centrifuged at 5000 rpm for 10 min, the supernatants were taken to determine their protein content and PTI inhibitory activity to calculate the protein and inhibitory activity recoveries of the inhibitors before and after sample solution treatment. This method was used only for preliminary comparisons of yield and activity; due to concerns about heavy metal residues, it is not included in subsequent characterization.

Ultrafiltration and freeze-drying of the samples were performed according to the method of Li et al. with slight modifications^13^. The sample solutions, obtained through various methods, were diluted to contain 1–2% solids. These diluted solutions were then concentrated using an ultrafiltration membrane with a molecular weight cut-off of 10 kDa. Following concentration, the samples were freeze-dried using a freeze-dryer to obtain the PTI sample.

### Characterization of PTI obtained by different preparation methods

FTIR was used to examine the structural composition of the product samples in order to make an in-depth comparison of the structural differences between the two product samples^57^. To prepare the test samples, 1 mg of the dried sample was blended with 100 mg of potassium bromide powder and compressed into tablets using a tablet press. These tablets were then positioned within the measurement cell for spectral analysis. The precise testing parameters were as follows: a wavelength scanning range spanning from 400 to 4000 cm^−1^, wavelength accuracy to 0.01 cm^−1^, a test resolution of 4 cm^−1^, and a cumulative scanning count of 64 scans. The entire testing procedure was conducted at a consistent temperature of 25 °C to guarantee the reliability of the results. The acquired IR spectral data was subjected to analysis using Ominc8.0 and Peakfit4.0 software, with a particular emphasis on the amide I band, located within the range of 1600~1700 cm^−1^.

The UV spectral detection method of the samples was referred to that of Yan et al.^58^ with slight modifications. The appropriate amount of sample was dissolved in deionized water to prepare a solution with a protein concentration of 100 μg/mL. The sample solution was scanned on a UV detector (Waters 2489, Waters Corporation, USA) under the following test conditions: scanning range of 200~400 nm, scanning speed of 600 nm/min, and data interval of 1 nm.

Surface hydrophobicity was determined according to Chen et al.^59^. After the products were diluted to step concentrations of 0.025 to 0.1 mg/mL of protein samples (0.01 M, pH 7 phosphate buffer), respectively, 4 mL of the protein sample solution was added with 20 μL of ANS (sodium 8-anilino-1-naphthalene sulfonate) fluorescent probe (8 mM) with sufficient mixing, and the solution was placed at room temperature to avoid the light and stood for the reaction for 3 min. The fluorescence intensity values of the samples were recorded at the excitation wavelength of 390 nm and emission wavelength of 470 nm, respectively, while phosphate buffer was used as a blank control group. The fluorescence intensity was used as the dependent variable, and the protein concentration was used as the independent variable to plot the graph, and the initial slope of the curve was the surface hydrophobicity.

The endogenous fluorescence intensity to each sample was measured according to the methodology outlined by Yu et al.^60^. The samples were diluted to a concentration of 0.1 mg/mL, and their fluorescence spectra were recorded at room temperature. The excitation wavelengths used were 258 nm, 275 nm, and 295 nm, respectively, while the emission spectra were detected within a range of 300–500 nm, with a slit width set to 5 nm.

The HORIBA SZ-100Z (Horiba, Japan) nanoparticle analyzer was utilized to assess the zeta potential, particle size distribution, and mean particle size of the samples^61^. The parameter configurations followed the guidelines provided by Baneshi et al.^62^ and the instrument’s test method database. Deionized water served as the dispersant, with inhibitor protein constituting the dispersed phase. The conditions for the zeta potential measurement were set at 25 °C and a refractive index of 1.330. Prior to testing, the protein samples were prepared into a 0.1% solution using deionized water and thoroughly mixed. These solutions were then transferred to the zeta potential measurement cuvette for testing. Each sample was tested in triplicate, and the average values were recorded.

### Solubility of PTI

The solubility of PTI was determined by referring to the method of Løkra et al. with minor modifications^14^. Protein solutions of 1% (w/v) were prepared by dissolving 40 mg of PTI solid powder in 4 ml of ultrapure water, respectively, and then the pH of the sample solutions was adjusted to 2, 4, 6, 8, 10, and 12 with 1 M HCl and 1 M NaOH solutions, respectively, at room temperature. The above solutions were shaken at 25 °C for 2 h in a 200-rpm shaker and then centrifuged at 8000 rpm for 20 min to collect the supernatant. The content of soluble proteins in the supernatant was determined by BCA kit method, and the total nitrogen in PTI solids was determined by Kjeldahl method, and the experiment was repeated three times for each sample. The solubility was calculated as in Formula (3):

3 $$\mathrm{Solubility}( \% )=\mathrm{S}/\mathrm{T}\times 100$$

Where *S* represents the soluble protein content in the supernatant (mg/L), *T* represents the total nitrogen content in the PTI (mg/L).

### Stability of PTI

The effect of different factors on the stability of PTI was carried out with reference to the modified method of Li et al.^13^. The prepared PTI (20 mg) was completely dissolved in 100 mL of 50 mM Tris-HCl buffer (pH 8.2). The resulting solution was divided evenly into five portions, and each group was placed in a water bath at 25, 45, 65, 85, and 100 °C for 10, 20, and 30 min, respectively. The inhibition rate of PTI in each group was determined by cooling to room temperature at the end of the holding period. The inhibition rate at 25 °C was set as the control group to calculate the retention rate of PTI inhibitory activity under different temperature and time conditions.

The prepared PTI (20 mg) was completely dissolved in 100 mL of 50 mM Tris-HCl buffer (pH 8.2). The solutions were equally divided into six groups, and their pH was adjusted to the corresponding 2, 4, 6, 8, 10, and 12 with 1 M HCl and 1 M NaOH, respectively. Subsequently, the pH was adjusted back to 8.2 after being kept at room temperature (25 °C) for 10, 20, and 30 min, respectively, to determine the percentage of inhibition of PTI in each group. The inhibition rate at pH 8.0 was set as a control group to calculate the retention of PTI inhibitory activity at different pH conditions.

The prepared PTI (40 mg) was completely dissolved in 200 mL of 50 mM Tris-HCl buffer (pH 8.2). The solutions were equally divided into 8 groups (one of which was a control group), and metal salts containing Na^+^, Cu^2+^, Ca^2+^, Mg^2+^, Zn^2+^, Mn^2+^, and Al^3+^ were added at room temperature (25 °C) to give a final concentration of 10 mM of each metal ion in the solution. The inhibition rate of PTI in each group was determined after 2 h of solution mixing. The inhibition rate under the condition without metal ions was set as a control group in order to calculate the retention of inhibitory activity of different metal ions on PTI.

The prepared PTI (40 mg) was completely dissolved in 200 mL of 50 mM Tris-HCl buffer (pH 8.2). Different amounts of Zn metal salts were added to the eight prepared PTI solutions at room temperature (25 °C) to achieve final concentrations of Zn^2+^ of 0, 0.5, 1, 2, 4, 6, 8, and 10 mM, respectively, and the inhibition rate of PTI in each group was determined after mixing for 2 h. Inhibition rates of the groups were determined using inhibition rates without the addition of Zn^2+^ as a control for calculating retention of inhibitory activities of PTI by the metal ions.

The prepared PTI (20 mg) was completely dissolved in 100 mL of 50 mM Tris-HCl buffer (pH 8.2). The three groups of prepared PTI solutions were kept at room temperature (25 °C) under daylight lamp, UV lamp and light-sheltered environment for 30 min, 1 h and 2 h, respectively, followed by determination of the retention inhibitory activity of each group of PTI.

The appropriate amount of PTI powder was taken out and divided into two groups; one group was exposed to air for 24 h, while the other group was kept in a sealed bag for 24 h. The solutions were prepared at the same concentration according to the method in 2.9.1, and the retention of inhibitory activity of PTI in the two groups was determined.

The in vitro simulated digestive stability of PTI was modeled after the previous method, with slight modifications^63^. For the simulated gastric digestion process, 60 mg of PTI powder was added to 300 mL of simulated gastric fluid blank (pH 2) and placed in a water bath shaker at 37 °C for 10 min with 200 rpm shaking. After adjusting the solution pH to 2.0 with 0.1 M HCl solution, 5 mL of freshly prepared simulated gastric fluid (pepsin concentration of 4 mg/mL) was added to it so that the ratio of pepsin to substrate mass concentration was 1:3. Subsequently, the mixture was re-placed in a shaker at 37 °C and 200 rpm for oscillatory reaction and sampled at different time points (0, 15, 30 and 60 min). Samples of 5 mL were taken at each time for determination of the degree of hydrolysis while another 1 mL was used to assess the inhibitory activity. After sampling, the pH of the samples was quickly adjusted to 6.8 by adding 1 M NaOH solution to terminate the reaction and centrifuged for 10 min (5000 rpm, 4 °C). Furthermore, a set of blank control groups, which contained only simulated gastric fluid blank without any substrate or enzyme added, and a set of sample control groups, which added substrate and simulated gastric fluid without pepsin, were set up separately.

For the simulated gastrointestinal digestion process, the pH of the simulated gastrointestinal digestion completed samples was adjusted to 6.8 by 1 M NaOH solution, followed by the sequential addition of 160 mL of simulated intestinal fluid blank (pH 6.8) and 6 mL of pre-warmed simulated intestinal fluid (pancreatin concentration of 10 mg/mL), to ensure that the ratio of pancreatin to the mass concentration of the substrate reached 1:1. The mixture was then placed into a 37 °C water bath shaker to oscillate the reaction at a rate of 200 rpm, and samples were taken at different time points (15 min, 30 min, 60 min, 120 min, 240 min) for analysis. The samples were centrifuged at 5000 rpm at 4 °C after sampling, and samples of 5 mL were taken each time for the determination of the degree of hydrolysis, and the other 1 mL was taken for the evaluation of the inhibitory activity. Meanwhile, two control groups were set up: the blank control group contained simulated gastric fluid and simulated intestinal fluid without adding substrate, while the sample control group contained substrate, blank gastric fluid, and blank intestinal fluid without adding pepsin and trypsin.

### Foaming properties and foam stability of PTI

Foaming properties and foam stability of PTI were carried out according to the method of Yang et al.^64^ with slight modifications. Potato simulated wastewater lyophilized powder and PTI were prepared into protein solutions of 1% (w/v) concentration at pH 2, 4, 6, 8, and 10, respectively, and the initial volume of the solution, *V*_0_, was recorded. Subsequently, the solution foam and total liquid volume *V*_1_ were recorded after being placed in a 25 °C water bath shaker oscillating at 200 rpm for 30 min and homogenized for 2 min using a high-speed dispersing homogenizer (T25, Germany IKA (Guangzhou) Instrument Co., Ltd) at 10,000 rpm, followed by the foam and total solution volume V_2_ obtained after the samples were allowed to stand for 30 min. Foaming and foam stability were calculated as shown in Formulas (4) and (5):

4 $$\mathrm{Foaming}( \% )=\frac{{\mathrm{V}}_{1}-{\mathrm{V}}_{0}}{{\mathrm{V}}_{0}}\times 100$$

5 $${\mathrm{Foam}}\,{\mathrm{stability}}( \% )=\frac{{\mathrm{V}}_{2}-{\mathrm{V}}_{0}}{{\mathrm{V}}_{1}{-\mathrm{V}}_{0}}\times 100$$

Besides, the freeze-dried powder of potato simulated wastewater and PTI protein solution were subjected to the above operation without pH adjustment, and the change of foam volume per minute was recorded during 90 min. Finally, the change curve was plotted with time as the horizontal coordinate and foam height change as the vertical coordinate.

### Emulsifiability and emulsion stability of PTI

The emulsifiability and emulsion stability of PTI were carried out according to the protocol used by Yu et al.^65^ with slight modifications. Two separate dispersions were prepared: a 1% (w/v) PTI dispersion and a 1% (w/v) potato waste concentrate freeze-dried powder dispersion (15 mL each). The pH of each dispersion was adjusted to 2, 4, 6, 8, or 10 using 1 M HCl or 1 M NaOH, followed by stirring at room temperature (25 °C) for 1 h. Subsequently, 10 mL of supernatant and 5 mL of vegetable oil were taken in a 50 mL beaker, and the mixture was homogenized using a high-speed dispersion homogenizer at 10,000 rpm for 2 min to obtain the sample emulsion. Samples of 20 μL of the bottom emulsion were taken at 0 min and 30 min, respectively, and mixed well with 10 mL of 0.1% sodium dodecyl sulfate (SDS) solution, followed by measuring the wavelength of the samples at 500 nm (A_500_) using a UV-visible spectrophotometer (UV 8000, Shanghai Yuan Analytical Instrument Co., Ltd), and a 0.1% SDS solution without emulsion was used as a blank control. The calculations of emulsifiability and emulsion stability are shown in Formulas (6) and (7):

6 $$\mathrm{Emulsifiability}\,({\rm{ \% }})=\frac{2.303\,\times \,2\,\times \,({\mathrm{A}}_{1}-{\mathrm{A}}_{0})\times \mathrm{N}}{\mathrm{L}\times \mathrm{c}\times (1-{\rm{\theta }})\times 10000}\times 100$$

7 $${\mathrm{Emulsion}}\,{\mathrm{stability}}\,( \% )=\frac{{\mathrm{A}}_{2}-{\mathrm{A}}_{0}}{{\mathrm{A}}_{1}{-\mathrm{A}}_{0}}\times 100$$

where *A* denotes the absorbance of the emulsion, 2.303 = ln (10), and 2 is the emulsion dilution factor, *N* denotes the dilution factor of the emulsion system (including factors such as the dilution factor of the protein solution and the volume of oil), and θ = 0.25, the oil phase volume fraction; *C* denotes the protein volume concentration, g/mL; L denotes the cuvette light range, 0.01 m; *A*_0_, *A*_1_ and *A*_2_ denote the absorbance at 0 min for the blank control and at 0 and 30 min for the sample, respectively.

### Data processing and statistical analysis

The analyzes of the data were carried out utilizing Origin 2024, Minitab 17, and GraphPad Prism 9.0. To assess the differences, ANOVA was used, followed by a Tukey post hoc test in SPSS 26. A statistically significant difference was indicated by a *p*-value < 0.05. To obtain the results, each experimental index was tested three times, and the data is presented as means ± SD.

## Supplementary information

Supplementary material

## Data Availability

The datasets generated and/or analyzed during the current study are not publicly available due to the fact that they are part of an ongoing research project and the corresponding author's institutional data management policies, but are available from the corresponding author on reasonable request.
