# Supplementary material for: Acid precipitation salting out as an efficient strategy for high purity potato trypsin inhibitor recovery
Source: NPJ Sci Food. 2026 Jun 2;10:299. doi: 10.1038/s41538-026-00912-6 (PMC13635096; doi:10.1038/s41538-026-00912-6)
Supplement: Supplementary file 1 [file 41538_2026_912_MOESM1_ESM.pdf]

Supplementary material

Acid precipitation salting out as an efficient strategy for high purity potato trypsin inhibitor recovery

Lei Cai [a, 1], Panchaonan Huang [b, 1], Chun Cui * [b]

[a] *School of Public Health and Health Management, Ganzhou Medical University, Ganzhou 341000, Jiangxi, China*

[b] *School of Food Science and Engineering, South China University of Technology, Guangzhou 510640, Guangdong, China*

[*] **Corresponding Authors**

Chun Cui——School of Food Science and Engineering, South China University of Technology, Guangzhou 510640, China. E-mail address: cuichun@scut.edu.cn (C. Cui); Fax: (+86) 20 87114954; Tel: (+86) 20 87114954

[1] These authors contribute equally to this work.

Figure Captions:

Figure S1 A: SDS-PAGE plots of potato wastewater after acid precipitation treatment (lane M: standard molecular proteins; lane 1: potato wastewater; lane 2: S2 (pH 2.0); lane 3: S2.5 (pH 2.5); lane 4: S3 (pH 3.0); lane 5: S3.5 (pH 3.5); lane 6: S4 (pH 4.0); lane 7: S4.5 (pH 4.5); lane 8: S5 (pH 5.0); B: SDS-PAGE plots of the supernatant after salting out treatment (lane M: standard molecular proteins; lanes 1~9: Y1~Y9); C: Effects of acid precipitation conditions (pH 2.0, 2.5, 3.0, 3.5, 4.0, 4.5, 5.0) on protein recovery and activity recovery from potato wastewater. Different letters indicate significant differences ($p < 0.05$); D: Effects of salting-out conditions on protein and activity recovery of potato waste liquid; E: SDS-PAGE plots of PTI prepared by acid precipitation combined with salting-out (lane M: standard molecular protein; lane 1: potato waste liquid; lane 2: PTI). Different letters indicate significant differences ($p < 0.05$).

Figure S2 A: Protein adsorption rate of different materials. **B:** Inhibitory activity adsorption rate. **C:** SDS-PAGE after adsorption. Lane M: marker; lanes 1&9: wastewater; 2: HPD82; 3: ADS-600; 4: AB-8; 5: LX-8; 6: HPD-400; 7: XDA-1; 8: LX-38; 10: HPD-600; 11: XAD-16; 12: LXTE-600; 13: LXTE-603; 14: hydrated silica. **D:** Protein desorption rate (different desorbents). **E:** Inhibitory activity recovery (different desorbents). **F:** SDS-PAGE after ethanol desorption. Lane M: marker; 1:20%; 2:40%; 3:60%; 4:80%; 5:100%.

Figure S3 A: Effect of different concentrations of Zn^{2+} on the recovery of inhibitory activity of potato wastewater; B: SDS-PAGE plots of the supernatant of potato wastewater after being precipitated by different concentrations of Zn^{2+} (lane M: standard molecular protein, lane 1: blank, lane 2: 0.5 mM Zn^{2+}, lane 3: 1 mM Zn^{2+}, lane 4: 2 mM Zn^{2+}, lane 5: 4 mM Zn^{2+}, lane 6: 6 mM Zn^{2+},

lane 7: 8 mM Zn^{2+}, and lane 8: 10 mM Zn^{2+}). Different letters indicate significant differences ($p < 0.05$).

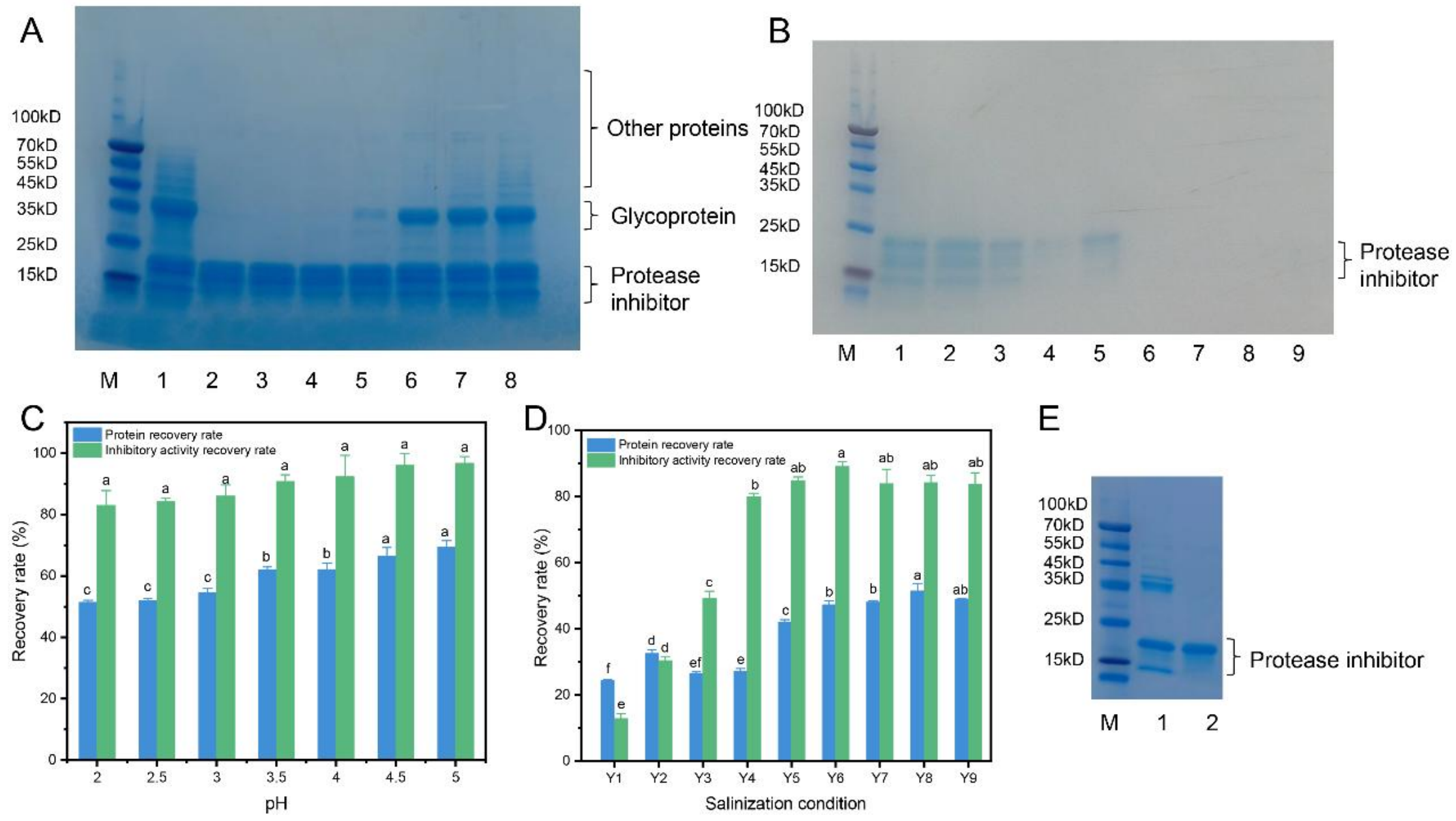

Figure S1 A: SDS-PAGE plots of potato wastewater after acid precipitation treatment (lane M: standard molecular proteins; lane 1: potato wastewater; lane 2: S2 (pH 2.0); lane 3: S2.5 (pH 2.5); lane 4: S3 (pH 3.0); lane 5: S3.5 (pH 3.5); lane 6: S4 (pH 4.0); lane 7: S4.5 (pH 4.5); lane 8: S5 (pH 5.0); B: SDS-PAGE plots of the supernatant after salting out treatment (lane M: standard molecular proteins; lanes 1~9: Y1~Y9); C: Effects of acid precipitation conditions (pH 2.0, 2.5, 3.0, 3.5, 4.0, 4.5, 5.0) on protein recovery and activity recovery from potato wastewater. Different letters indicate significant differences ($p < 0.05$); D: Effects of salting-out conditions on protein and activity recovery of potato waste liquid; E: SDS-PAGE plots of PTI prepared by acid precipitation combined with salting-out (lane M: standard molecular protein; lane 1: potato waste liquid; lane 2: PTI). Different letters indicate significant differences ($p < 0.05$).

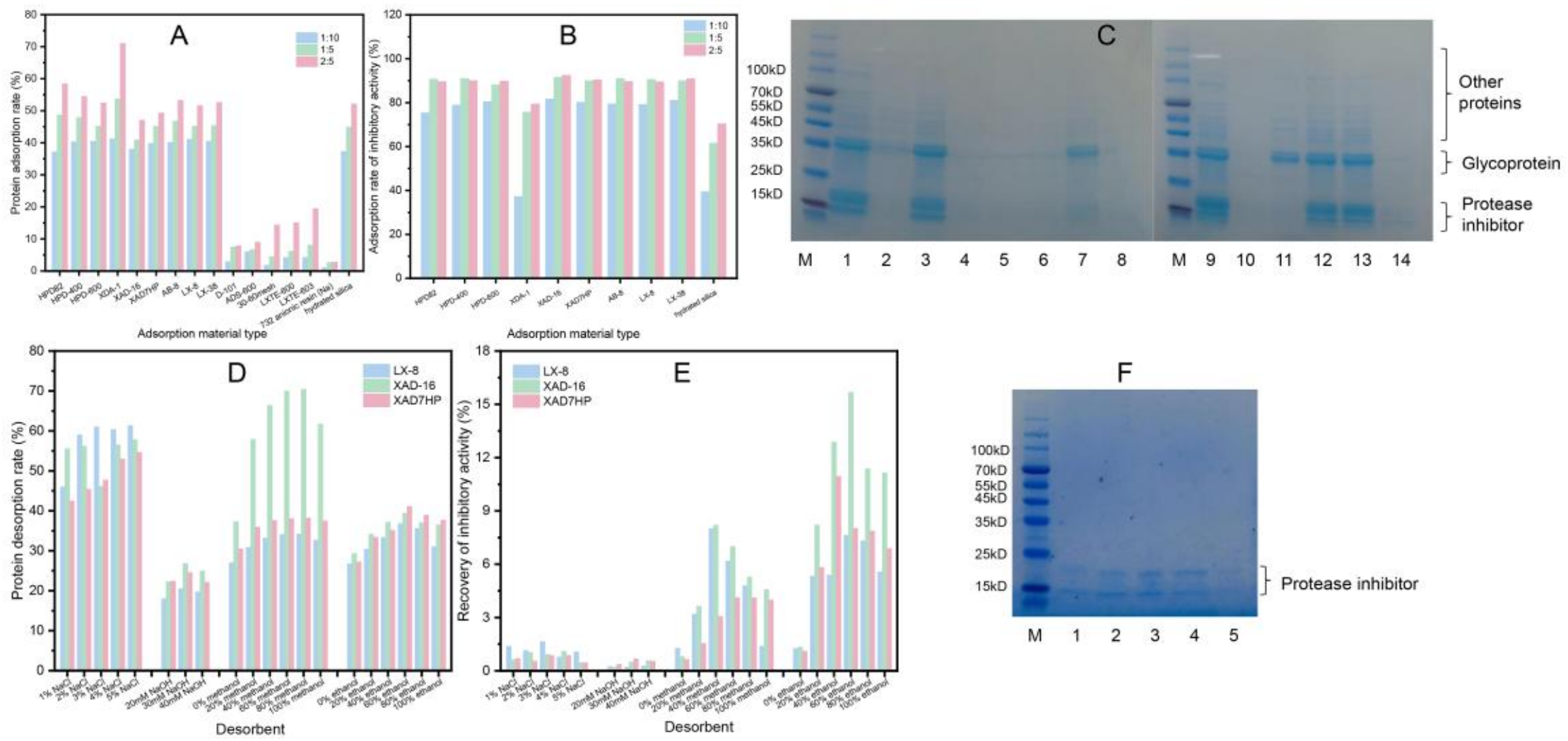

Figure S2 A: Protein adsorption rate of different materials. **B:** Inhibitory activity adsorption rate. **C:** SDS-PAGE after adsorption. Lane M: marker; lanes 1&9: wastewater; 2: HPD82; 3: ADS-600; 4: AB-8; 5: LX-8; 6: HPD-400; 7: XDA-1; 8: LX-38; 10: HPD-600; 11: XAD-16; 12: LXTE-600; 13: LXTE-603; 14: hydrated silica. **D:** Protein desorption rate (different desorbents). **E:** Inhibitory activity recovery (different desorbents). **F:** SDS-PAGE after ethanol desorption. Lane M: marker; 1:20%; 2:40%; 3:60%; 4:80%; 5:100%.

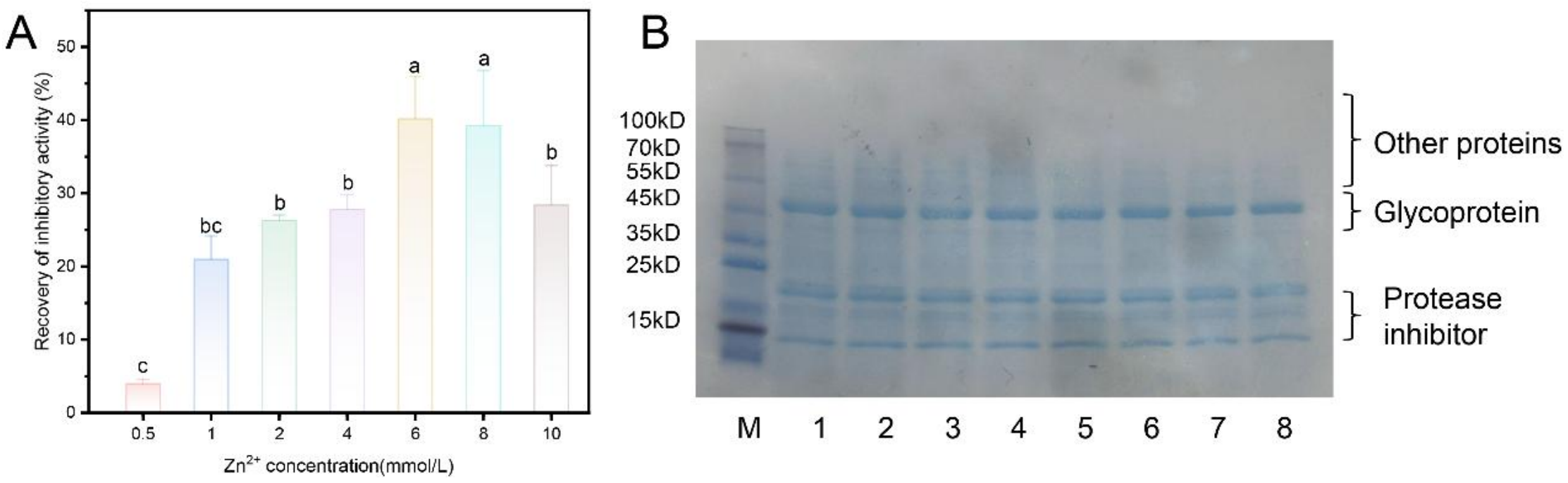

Figure S3 A: Effect of different concentrations of Zn^{2+} on the recovery of inhibitory activity of potato wastewater; B: SDS-PAGE plots of the supernatant of potato wastewater after being precipitated by different concentrations of Zn^{2+} (lane M: standard molecular protein, lane 1: blank, lane 2: 0.5 mM Zn^{2+}, lane 3: 1 mM Zn^{2+}, lane 4: 2 mM Zn^{2+}, lane 5: 4 mM Zn^{2+}, lane 6: 6 mM Zn^{2+}, lane 7: 8 mM Zn^{2+}, and lane 8: 10 mM Zn^{2+}). Different letters indicate significant differences ($p < 0.05$).

Table Captions：

Table S1 Protein and total sugar content in simulated potato processing wastewater.

Table S2 Zinc ion (Zn^{2+}) content of each component after precipitation.

Table S3 Particle size of samples obtained by different methods.

Table S1 Protein and total sugar content in simulated potato processing wastewater.

Index	Content (%)
Protein	0.53±0.02
Total sugar	1.30±0.06
Solids	2.57

Table S2 Zinc ion (Zn^{2+}) content of each component after precipitation.

Sample	Potato wastewater	Precipitation supernatant	Precipitation freeze-dried powder
Zinc ion content (Zn^{2+}) (mg/kg)	1.37	1253.83	41599.05

Table S3 Particle size of samples obtained by different methods.

Samples	Acid precipitation-salting out	Direct adsorption
d average/nm	346.2±2.1	783.8±2.7
d (0.05)/nm	317.9	463.0
d (0.1)/nm	320.5	498.1
d (0.5)/nm	342.2	731.1
d (0.9)/nm	383.6	1155.1
